# A Guide to Conducting a Meta-Analysis with Non-Independent Effect Sizes

**DOI:** 10.1007/s11065-019-09415-6

**Published:** 2019-08-24

**Authors:** Mike W.-L. Cheung

**Affiliations:** Department of Psychology, Faculty of Arts and Social Sciences, National University of Singapore, Block AS4, Level 2, 9 Arts Link, Singapore, 117570 Singapore

**Keywords:** Meta-analysis, Multivariate meta-analysis, Three-level meta-analysis, Non-independent effect size

## Abstract

Conventional meta-analytic procedures assume that effect sizes are independent. When effect sizes are not independent, conclusions based on these conventional procedures can be misleading or even wrong. Traditional approaches, such as averaging the effect sizes and selecting one effect size per study, are usually used to avoid the dependence of the effect sizes. These ad-hoc approaches, however, may lead to missed opportunities to utilize all available data to address the relevant research questions. Both multivariate meta-analysis and three-level meta-analysis have been proposed to handle non-independent effect sizes. This paper gives a brief introduction to these new techniques for applied researchers. The first objective is to highlight the benefits of using these methods to address non-independent effect sizes. The second objective is to illustrate how to apply these techniques with real data in R and Mplus. Researchers may modify the sample R and Mplus code to fit their data.

**Electronic supplementary material:**

The online version of this article (10.1007/s11065-019-09415-6) contains supplementary material, which is available to authorized users.

A single study rarely provides enough evidence to address research questions in a particular domain. Replications are generally the preferred approach for addressing critical scientific questions (e.g., Open Science Collaboration, 2012, 2015). Replications of studies are particularly important, given that many of the published findings are said to be non-replicable. When there is a large pool of empirical studies on a similar topic, a meta-analysis can be used to synthesize these research findings (Anderson & Maxwell, 2016). Meta-analysis is generally recognized as *the* method for synthesizing research findings in disciplines across the social, behavioral, and medical sciences (e.g., Gurevitch, Koricheva, Nakagawa, & Stewart, 2018; Hedges & Schauer, 2018; Hunt, 1997). A few psychological journals, such as *Psychological Bulletin* (Albarracín et al., 2018) and *Neuropsychology Review* (Loring & Bowden, 2016), are dedicated to publishing high-quality systematic reviews and meta-analyses.

Many books introducing how to conduct a systematic review and meta-analysis have already been published (e.g., Borenstein, Hedges, Higgins, & Rothstein, 2009; Card, 2012; Cheung, 2015a; Cooper, Hedges, & Valentine, 2009; Hedges & Olkin, 1985). Cheung and Vijayakumar (2016) recently gave a brief introduction to how neuropsychologists can conduct a meta-analysis. Their introduction assumes that the effect sizes are independent, which is a crucial assumption in a meta-analysis. It is rare for primary studies to report only one relevant effect size. Reported effect sizes are likely to be non-independent for various reasons. The sampling errors of the effect sizes may be correlated because the same participants are involved in calculating the effect sizes. For example, the same control group is used in calculating the treatment effects or there is more than one outcome effect size. Another reason for non-independent effect sizes is that the effect sizes of the independent samples are nested within a primary study. This nested structure will create dependence when a meta-analysis is conducted. Results based on conventional meta-analytic methods are inappropriate or even misleading. Many advances in how to handle non-independent effect sizes have been made in the past decade. Applied researchers, however, may not be familiar with these advanced meta-analytic techniques.

Therefore, the primary objective of this paper is to give an introduction on how to handle non-independent effect sizes in a meta-analysis. We will introduce the multivariate meta-analysis and three-level meta-analysis to handle two types of non-independence in a meta-analysis. The second objective is to illustrate how to apply these techniques with real data in the R statistical platform (R Development Core Team, 2019) and Mplus (Muthén & Muthén, 2017). Researchers may modify the sample R and Mplus code to fit their models. In the following sections, we first provide some background on the problems arising from non-independent effect sizes and how to address these problems with conventional versus preferred meta-analytic methods. Two real examples in published meta-analyses are used to illustrate how to analyze non-independent effect sizes.

## What Are the Key Assumptions in a Meta-Analysis?

To facilitate the introduction, we first review a standard random-effects meta-analytic model (e.g., Borenstein et al., 2009; Hedges & Olkin, 1985). We use *y*_*i*_ to represent a generic effect in the *i*th study. The effect size can be either a standardized (or raw) mean difference, a correlation coefficient (or its Fisher’s z transformation), a log-odds ratio, or some other effect size (e.g., Cheung & Vijayakumar, 2016). The random-effects meta-analytic model is:

1 $$ {y}_i={\beta}_R+{u}_i+{e}_i, $$

where *β*_*R*_ is the average population effect, *Var*(*u*_*i*_) = *τ*^2^ is the population heterogeneity variance that has to be estimated, and *Var*(*e*_*i*_) = *v*_*i*_ is the known sampling variance in the *i*th study. The heterogeneity variance *τ*^2^ is an absolute index of heterogeneity that depends on the type of effect size. That is, we cannot compare the computed heterogeneity variances across different types of effect size. We may calculate a relative heterogeneity index *I*^2^ to indicate what percentage of the total heterogeneity is comprised by between-study heterogeneity (Higgins & Thompson, 2002). It should be noted that the value of *I*^2^ is affected by the “typical” within-study sampling variance, which is affected by the sample sizes in the primary studies (Borenstein, Higgins, Hedges, & Rothstein, 2017). Given the same value of *τ*^2^, *I*^2^ may become larger when the “typical” within-study sampling variance becomes smaller.

When there is excess variation in the population effect sizes, researchers may want to explain the heterogeneity in terms of the characteristics of the study. The model can be extended to include moderators, say *x*_*i*_, to explain the heterogeneity of the effect sizes:

2 $$ {y}_i={\beta}_0+{\beta}_1{x}_i+{u}_i+{e}_i, $$

where *β*_0_ and *β*_1_ are the intercept and regression coefficient, respectively. Multiple moderators may be included in the model. When there is a categorical moderator with more than two categories, dummy coded moderators may be used. In addition to testing the significance of the moderators, we may also calculate an *R*^2^ index to quantify the percentage of the heterogeneity variance that can be explained by adding the moderators.

There are two critical assumptions in random- and mixed-effects meta-analyses. First, the sample effect size *y*_*i*_ is conditionally distributed as a normal distribution with a known sampling variance *v*_*i*_. Several factors may affect the appropriateness of this assumption. The first factor is the type of the effect size. A raw mean difference, for example, approaches a normal distribution much faster than would a correlation coefficient or an odds ratio. For a correlation coefficient and an odds ratio, we may apply transformations to “normalize” their sampling distributions. For example, a log transformation on the odds ratio and a Fisher’s z transformation on the correlation coefficient are usually applied before a meta-analysis is conducted. Another factor is the size of the sample. If the sample size is large enough, the sampling variance of the effect size can be assumed to be approximately normal and known. Depending on the types of effect sizes, reasonably large sample sizes in primary studies are expected in a meta-analysis. Some (transformed) effect sizes, for example, the raw mean difference and the Fisher’s z transformed score, work well even for small sample sizes when the underlying populations are normally distributed.

The second critical assumption is that the effect sizes are independent. When the effect sizes in a meta-analysis are not independent, the estimated standard errors (*SE*s) on the average effect are generally under-estimated (López-López, Van den Noortgate, Tanner-Smith, Wilson, & Lipsey, 2017). Researchers may incorrectly conclude that the average effect is very precise. This problem is well known in the context of multilevel models (Goldstein, 2011; Hox, 2010; Raudenbush & Bryk, 2002). If we incorrectly treat the non-independent data as independent, the statistical inferences are likely to be wrong. Therefore, researchers should not treat non-independent effect sizes as if they were independent. How to handle non-independent effect sizes is the focus of this paper.

## How Many Types of Non-Independent Effect Sizes Are There?

We may roughly classify non-independent effect sizes into multivariate and nested effect sizes. Other more sophisticated types of non-independence will be addressed in the Conclusion and Future Directions section. Table 1 shows a sample data structure of two multivariate effect sizes. *y*_1_ and *y*_2_ represent two different outcome measures, for example, physical and psychological improvements after a treatment. Both *y*_1_ and *y*_2_ are reported in Study 1, whereas only one of the two is reported in Studies 2 and 3.

**Table 1:** Sample data structure for a multivariate meta-analysis with two multivariate effect sizes

Study	*y*_1_	*y*_2_	*V*_11_	*V*_21_	*V*_22_
1	.35	.52	.02	.01	.02
2	.43	NA	.03	NA	NA
3	NA	.27	NA	NA	.01

*y*_1_ and *y*_2_ are the multivariate effect sizes. *V*_11_, *V*_21_, and *V*_22_ are the known sampling variances and covariance of *y*_1_ and *y*_2_. NA represents not available

With regard to multivariate effect sizes, the sampling errors of the effect sizes are usually conditionally correlated because the same participants are used when calculating the multiple effect sizes (e.g., Raudenbush, Becker, & Kalaian, 1988; Timm, 1999). In Table 1, *V*_21_ represents the sampling covariance of *y*_1_ and *y*_2_, which is usually non-zero. For example, the common practice is to have several treatment groups with one control group in experimental or intervention studies. The effect sizes of the treatment groups are calculated against the same control group. The effect sizes in this setting are non-independent because the same control group is used to calculate the effect sizes. Studies that employ this approach are known as multiple-treatment studies (Gleser & Olkin, 2009).

A second example of multivariate effect sizes is the multiple-endpoint study (Gleser & Olkin, 2009). Abramovitch, Anholt, Raveh-Gottfried, Hamo, and Abramowitz (2018) investigated the effects of Obsessive Compulsive Disorder (OCD) on Intelligence Quotient (IQ). Since IQ scores may be assessed in terms of Full-Scale IQ, Verbal IQ, and Performance IQ, the effect can be conceptualized as three inter-related outcomes. The degree of dependence of the multivariate effect sizes, *V*_21_ in Table 1, may be calculated from the summary statistics (Cheung, 2018; Gleser & Olkin, 2009).

A third example is drawn from the study of Weissberger et al. (2017), who were interested in examining the accuracy of neuropsychological assessments in detecting mild cognitive impairment (MCI) and Alzheimer’s dementia (AD). The accuracy of such assessments is usually quantified by the sensitivity and specificity of the tests that are used to make the assessment. The sampling errors of the sensitivity and specificity are conditionally independent because there is no overlapping of participants in the groups with and without condition (or disease; e.g., Li & Fine, 2011). The random effects, however, may still be correlated. Therefore, we should still treat the sensitivity and specificity as multivariate effect sizes in the analysis.

The second type of dependence is attributable to nested effect sizes, that is, the effect sizes that are nested within a unit, for example, a study. Table 2 displays a sample data structure of nested effect sizes. The label “Cluster” indicates how the independent effect sizes are grouped together. The sampling error *v* of the effect size *y* is conditionally independent. Thus, there is no sampling covariance *V*_21_ in the data structure. Since the effect sizes within a cluster are likely to be more similar to each other than the effect sizes across clusters, the population effect sizes may not be independent. This situation is similar to the case of participants nested within a level-2 unit in a multilevel model. A study may report multiple effect sizes from multiple independent samples. These effect sizes are measuring the same construct relevant to our research questions, namely, that it is fine to combine these effect sizes into a single effect size. For example, Mauger et al. (2018) studied how the Turner syndrome affected executive functions in children and adolescents. Since more than one effect sizes on the executive functions were reported in the primary studies, these authors treated the effect sizes as nested within the primary studies.

**Table 2:** Sample data structure for a three-level meta-analysis

Cluster	*y*	*v*
1	.32	.02
1	.54	.02
1	.41	.01
2	.06	.03
2	.02	.03
3	.37	.05

*y* is the effect size and *v* is the known sampling variance of *y*. Cluster indicates how the effect sizes are grouped

Another example is that we may conceptualize some higher-level units, for example, country or research groups, as our unit of analysis. The reported effect sizes (or studies) are nested within these higher-level units. There are several such examples in cross-cultural meta-analyses, where the studies are nested within the countries (Fischer & Boer, 2011; Fischer, Hanke, & Sibley, 2012). Another interesting example is the single-subject design. In single-subject designs, effect sizes are calculated for each subject. The effect sizes of the subjects are nested within studies. When researchers meta-analyze these effect sizes, they have to take the dependence of the effect sizes into account (Moeyaert, Ugille, Ferron, Beretvas, & Van den Noortgate, 2013).

## What Are the Common Approaches to Handling Multivariate Effect Sizes?

We use the example of Abramovitch et al. (2018), which has been introduced, to start our discussion. These authors extracted 98 studies containing the IQ scores of OCD patients and non-psychiatric comparison groups. Since the primary studies reported some of the Full Scale IQ, Verbal IQ, and Performance IQ scores, three separate effect sizes might be calculated in each study. One popular option for dealing with multivariate effect sizes is to analyze them independently. Abramovitch et al. (2018) conducted three separate meta-analyses on Full Scale IQ, Verbal IQ, and Performance IQ. There are several such examples in the literature (e.g., Belleville et al., 2017; Weissberger et al., 2017). This approach is appealing because no new technique needs to be used. However, the primary limitation of this approach is that it does not take into account in the analyses the advantage arising from the dependence of the effect sizes.

A multivariate meta-analysis is generally recommended for handling multivariate effect sizes (e.g., Cheung, 2013; Hedges & Olkin, 1985; Nam, Mengersen, & Garthwaite, 2003; Raudenbush et al., 1988; Jackson, Riley, & White, 2011). In this approach, the idea is similar to extending an ANOVA to a MANOVA to handle more than one dependent variable. Now, let ***y***_*i*_ be a vector of *p* × 1 effect sizes (*p* is the number of outcome effect sizes). The meta-analytic model in Eq. (1) can be extended to handle multivariate effect sizes as follows:

3 $$ {\boldsymbol{y}}_i={\boldsymbol{\beta}}_R+{\boldsymbol{u}}_i+{\boldsymbol{e}}_i, $$

where ***β***_*R*_ is the vector of the average population effects, *Cov*(***u***_*i*_) = ***T***^2^ is the *p* × *p* population heterogeneity variance-covariance matrix that has to be estimated, and *Cov*(***e***_*i*_) = ***V***_*i*_ is the *p* × *p* known sampling variance-covariance matrix in the *i*th study that is computed from the summary statistics (Cheung, 2015a, Chapter 3). When the studies report different numbers of effect sizes, the incomplete effect sizes are filtered out before the analysis. This equation can easily be extended to a mixed-effects model, as we did in Eq. (2).

Apart from estimating the average population effects ***β***_*R*_ and their heterogeneity variance-covariance matrix ***T***^2^, several interesting research questions can be tested in a multivariate meta-analysis. Using the study of Abramovitch et al. (2018) as an example, we may treat the IQ domains (Full Scale IQ, Verbal IQ, and Performance IQ) as multiple outcomes and compare whether the average means of the OCD patients of these IQ domains are the same. We may also verify whether the heterogeneity variances are the same across different IQ domains. By inspecting the means and heterogeneity variances, researchers may get a better idea of what the effect of the OCD is. Moreover, we may study the correlation between the population random effects (IQ domains in our example). If the correlation is high, this indicates that studies with a higher population effect on one IQ domain are associated with studies with a higher population effect on another IQ domain.

When comparing the univariate and multivariate meta-analyses, Ishak, Platt, Joseph, and Hanley (2008) have argued that researchers may conduct univariate meta-analyses without introducing any bias or loss of precision in the fixed-effects estimates. Several authors (e.g., Demidenko, 2013; Riley, 2009) have shown that the estimated fixed effects in a multivariate meta-analysis usually have smaller *SE*s. In other words, we may get more precise estimates (smaller confidence intervals (CIs)) by using a multivariate meta-analysis.

Two factors may affect the usefulness of multivariate meta-analyses. The first factor is the correlation between the population effect sizes. The presence of a positive (or negative) association between the effect sizes reduces the uncertainty of the estimates of other effect sizes. This is similar to the case of the MANOVA (see Cheung, 2015a, Section 5.1.2 for a discussion). The second factor is the number of studies with complete effect sizes. If there are only a few studies with complete effect sizes, there would be no information to estimate the correlation among the population effect sizes. Suppose that all of the primary studies only report either the Full Scale IQ, Verbal IQ, or Performance IQ, then the estimated correlation between the population effect sizes would be zero. Should there be no correlation among the population effect sizes, the results of the univariate and multivariate meta-analyses would be the same. Researchers are encouraged to apply a multivariate meta-analysis whenever possible (Jackson et al., 2011) because of the benefits that can be obtained from the correlated effect sizes. In the worst-case scenario where the effect sizes are uncorrelated, the results of the multivariate meta-analysis would be similar to that which would be obtained from running several univariate meta-analyses.

## What Are the Common Approaches to Handling Nested Effect Sizes?

When the effect sizes are nested within some hierarchies, for example, studies, there is a clear consensus that we should not ignore the dependence and analyze the data as if they were independent. If we ignore the dependence, the *SE*s and the statistical inferences of the analyses would likely be incorrect.

A three-level meta-analysis was proposed to address the problems mentioned above (Cheung, 2014; Konstantopoulos, 2011). The standard meta-analytic model in Eq. (1) can be extended to handle nested effect sizes. We use *y*_*ij*_ to represent the *i*th effect size in the *j*th study. The three-level meta-analysis is:

4 $$ {y}_{ij}={\beta}_R+{u}_{(2) ij}+{u}_{(3)j}+{e}_{ij}, $$

where *β*_*R*_ and *e*_*ij*_ are similarly defined in Equation (1), and $Var\left({u}_{(2) ij}\right)={\tau}_{(2)}^2$ and $Var\left({u}_{(3)j}\right)={\tau}_{(3)}^2$ are the level-2 and level-3 heterogeneity variances, respectively. This analysis can easily be extended to a mixed-effects model, as we did in Equation (2).

There are several advantages to applying this three-level meta-analysis on nested effect sizes. First and most important, the level-2 heterogeneity variance ${\tau}_{(2)}^2$ takes the dependence into account in the analyses. Results based on a conventional meta-analysis and a three-level meta-analysis are identical only when the level-2 heterogeneity variance is zero. Second, researchers may study the level-2 and level-3 heterogeneity variances and their *I*^2^ counterparts at level-2 and level-3. Third, researchers may also investigate how the level-2 and level-3 moderators explain the heterogeneity using *R*^2^ at level-2 and level-3. These additional statistical analyses allow researchers to study the heterogeneity at different levels (see Cheung, 2014).

Before leaving this section, we have to mention another procedure that is used to handle dependent effect sizes. It is called the robust variance estimation (Hedges, Tipton, & Johnson, 2010; Tipton, 2015). Instead of estimating the dependence with the level-2 heterogeneity variance with Equation (4), this approach ignores the dependence by calculating an adjusted *SE*. One advantage of the robust variance estimation is that it can be applied to both the multivariate and nested effect sizes (see the discussion in the next section). On the other hand, the three-level meta-analysis allows researchers to study the heterogeneity variances *τ*^2^ (and *R*^2^) at different levels, whereas the robust variance estimation combines these effects into one single value.

## What Are the Relationships between a Multivariate Meta-Analysis and a Three-Level Meta-Analysis?

It is of importance to clarify some key similarities and differences between a multivariate meta-analysis and a three-level meta-analysis. A multivariate meta-analysis is conducted when the sampling covariances are known. That is, the sampling errors are not independent because the same participants are used in calculating the effect sizes. For example, multiple-treatment and multiple-endpoint studies are typical applications of multivariate meta-analysis.

On the other hand, a three-level meta-analysis has another set of assumptions. The typical application of a three-level meta-analysis is the scenario where reported effect sizes are nested within studies. The participants only contribute to one effect size, that is, there are no repeated measures. Thus, the sampling errors in a three-level meta-analysis are conditionally independent. The non-independence is primarily introduced due to the nested structure of the effect sizes.

Technically speaking, multivariate effect sizes are also nested within studies. We may arrange the multivariate effect sizes in Table 1 to the nested structure in Table 2. The only uncertain part is how to handle *V*_21_ because the sampling variances are assumed to be independent in the nested effect sizes in Table 2 (see Cheung, 2013, and Raudenbush et al., 1988 on how to transform correlated effect sizes into independent effect sizes). Mathematically, these two models are closely related. A three-level meta-analysis can be formulated as a special case of a multivariate meta-analysis, whereas a multivariate meta-analysis can be approximated by a three-level meta-analysis with some additional assumptions (see Cheung, 2015a, Section 6.4 for the details). Researchers may think carefully which technique, whether a multivariate meta-analysis or a three-level meta-analysis, is the most appropriate to use to analyze the data.

Multivariate effect sizes are probably more common than nested effect sizes in applications of meta-analysis. One main difficulty of applying a multivariate meta-analysis is the requirement to calculate the sampling covariances among the effect sizes. When these correlations are not available, several options are available to deal with this situation (e.g., Riley, Thompson, & Abrams, 2008). One popular approach is to average the effect sizes within a study and use this figure in subsequent meta-analyses (Borenstein et al., 2009). Averaging the effect sizes within a study is easy. There are several such examples of this approach in the literature (e.g., Burmester, Leathem, & Merrick, 2016; Mewborn, Lindbergh, & Stephen Miller, 2017; Sherman, Mauser, Nuno, & Sherzai, 2017). However, it is less straightforward to calculate the sampling variances of the average effect sizes. In calculating the sampling variances of the average effect sizes, we need to know the correlations among the effect sizes. Published studies rarely provide information that can be used to estimate these correlations. Researchers usually use either 0 or 1 or some arbitrary values in the calculations. The assumed value of the correlation may affect the subsequent meta-analyses. Researchers may check the sensitivity of the results by using a range of possible correlations. The essential idea of a sensitivity analysis is to investigate whether the conclusions would be different if a different value of correlation is used in the calculations. If the conclusions are the same, the findings are robust to the values of the correlation and researchers do not need to worry about the stability of the findings. On the other hand, researchers have to interpret the results with caution when the conclusions vary a great deal and depend on the values of the correlation.

Another popular alternative is to select one effect size from the available effect sizes within a study. Several variations have been used in choosing the effect sizes for meta-analyses. Some researchers randomly choose one effect size per study, while others may provide reasons for selecting a particular scheme. For example, they may choose “popular” measures or measures with better psychometric properties. If the effect sizes are randomly chosen, the average effect is unbiased. However, the estimates are less efficient because some effect sizes have been dropped. If there is a selection scheme, for example, choosing the more popular measures, the results may be biased towards these measures. This is because studies using the most popular measures may not represent the studies published in the literature.

There are several other limitations to these approaches. First, they do not utilize all the available data. It is generally difficult and expensive to extract effect sizes form the source literature for a meta-analysis. Averaging or selecting one effect per study means that much valuable information has to be removed from the analyses. Second, averaging the effect sizes or selecting one effect size within a study may remove valuable within-study variations stemming from potential moderators. For example, the effect sizes within a study may represent different types of measures and conditions. If we average these effect sizes into a single value or only select one effect size, it would not be possible to study whether the measures and conditions are moderating the effect.

Another option is to treat the multivariate effect sizes as the nested effect sizes in a three-level meta-analysis. Dummy codes are used to represent different effect sizes. For example, we may treat effect sizes on the Full Scale IQ, Verbal IQ, and Performance IQ in a multivariate meta-analysis as nested effect sizes in a three-level meta-analysis. We may then include dummy codes to represent the effect sizes. By making a few additional assumptions, we may analyze a multivariate meta-analysis as a three-level meta-analysis without knowing the sampling covariances of the multivariate effect sizes (see Cheung, 2015a, Section 6.4.2). Computer simulations (e.g., Moeyaert et al., 2017; Van den Noortgate, López-López, Marín-Martínez, & Sánchez-Meca, 2013) usually suggest that this approach works reasonably well under simulated conditions. Since it is quite likely that the correlations among the effect sizes are missing in the meta-analyses, many researchers prefer the three-level meta-analysis to the multivariate meta-analysis.

Alternatively, the robust variance estimation (Hedges et al., 2010; Tipton, 2015) can also be applied to effect sizes with correlated sampling errors where the sampling covariances are not available. Simulation studies have shown that both the three-level meta-analysis and the robust variance estimation work very well in simulated conditions (Moeyaert et al., 2017).

## How to Conduct a Multivariate Meta-Analysis and a Three-Level Meta-Analysis?

The metaSEM (Cheung, 2015b) and metafor (Viechtbauer, 2010) packages implemented in the R statistical platform can be used to conduct multivariate and three-level meta-analyses. Mplus may also be used to perform these analyses (Cheung, 2015a, Chapter 9). In this paper, we will illustrate the analyses of the multivariate meta-analysis and three-level meta-analysis with the R statistical platform and Mplus. The data are available in the metaSEM package, whereas the complete R code and the output of the analyses are shown in the Supplementary Materials. Readers may easily reproduce and replicate the results. It should be noted that the analyses here are meant to illustrate the procedures of the multivariate and three-level meta-analyses. The data and results may be slightly different from the ones used in the original meta-analyses because the data were obtained from their published tables rather than directly from the authors of the meta-analyses. Readers interested in the substantive research questions may refer to the original meta-analyses.

### Multivariate Meta-Analysis

The sample data were adopted from Table 1 of Nam et al. (2003), who studied the effects of environmental tobacco smoke, or passive smoking, on the health of children. The effect sizes used in the analyses were the log-odds ratios of the group with environmental tobacco smoke against a normal control group in the development of asthma and lower respiratory disease. Since the correlation between asthma and lower respiratory disease was not available in the paper, we used a correlation of 0.5 to calculate the sampling covariance between the effect sizes. A sensitivity analysis was also conducted by using a correlation of 0 and .8.

There are a total of 59 studies in the data set “Nam03” in the metaSEM package. Eight of these studies include both asthma and lower respiratory disease, while the remaining studies only include one of these two effect sizes. If we conduct two separate meta-analyses, the average effects (and their *SE*s) on asthma and lower respiratory disease are 0.23 (0.05) and 0.30 (0.06), respectively. The estimated heterogeneity variances on asthma and lower respiratory disease are 0.04 and 0.05, respectively. The estimated *I*^2^ on asthma and lower respiratory disease are 0.73 and 0.92, respectively.

The results of the multivariate meta-analysis on asthma and lower respiratory disease are 0.27 (0.05) and 0.31 (0.05), respectively. The estimated heterogeneity variances on asthma and lower respiratory disease are 0.07 and 0.05, respectively. The estimated *I*^2^ on asthma and lower respiratory disease are .82 and .92, respectively. The results of the univariate and multivariate meta-analyses are comparable in this case. However, there is no guarantee that the estimated *SE*s will be similar. It all depends on the data.

**Fig. 1:**
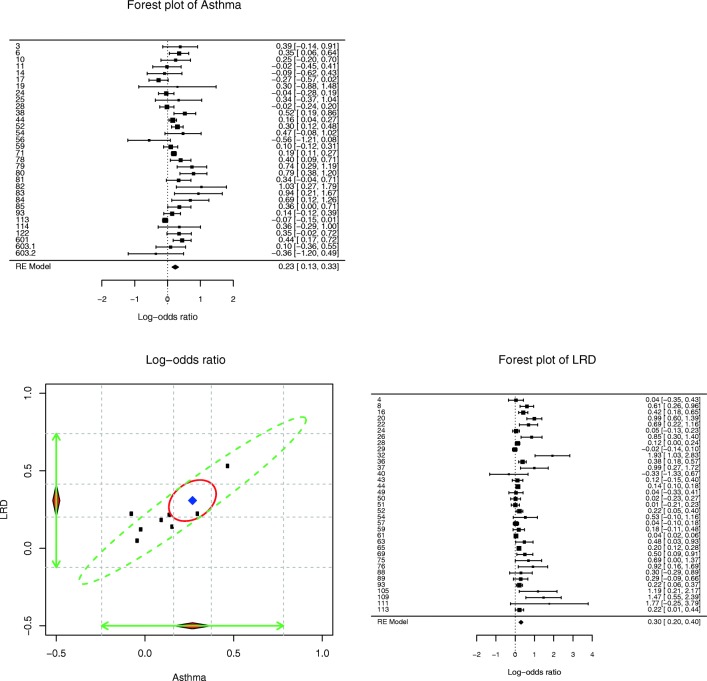
Plot of multivariate effect sizes and forest plots

We may take advantage of the multivariate meta-analysis by testing several additional research questions. First, the estimated correlation between the random effects is .96, which suggests that studies with a large effect on asthma tend to be associated with studies with a large effect on lower respiratory disease. Figure 1 shows the forest plots on asthma and lower respiratory disease and the 95% confidence ellipses on the average effects (red solid ellipse) and the studies (green dashed ellipse). Ninety-five percent of the studies likely fall into the green dashed ellipse. Because of the high correlation between the random effects (.96), we are more certain about the position of the studies. If we had only conducted two separate univariate meta-analyses, we would not have known that the effects of asthma and lower respiratory disease are highly correlated.

In a multivariate meta-analysis, we may test whether the average effects on asthma and lower respiratory disease are the same and whether their heterogeneity variances are also the same. Comparing the models with and without these two constraints on the means and variances, the *χ*^2^(*df* = 2) = 2.78, *p* = .25. Therefore, there is no evidence to reject the null hypothesis that the effects are the same in asthma and lower respiratory disease.

We may further conduct a mixed-effects multivariate meta-analysis by using the mean age of the participants as a moderator. The estimated regression coefficients on asthma and lower respiratory disease and their *SE*s are −0.04 (0.02), *p* = .01 and − 0.02 (0.01), *p* = .01, respectively. Their *R*^2^ are .59 and .39, respectively. The effect of environmental tobacco smoke is weaker in studies with older participants. Similarly, we may also test whether the regression coefficients on asthma and lower respiratory disease are the same. By comparing the models with and without the constraint on the regression coefficients, the *χ*^2^(*df* = 1) = 0.64, *p* = .42. Therefore, there is no evidence to reject the null hypothesis that the moderating effect of the mean age of the participants is the same in asthma and lower respiratory disease.

In the above analyses, we used a correlation of .5 to calculate the sampling covariances between the effect sizes of asthma and lower respiratory disease. We conducted a sensitivity analysis using a correlation of 0 and .8. The results were very similar. Therefore, our results are robust to the choices of the correlation in calculating the sampling covariances between the effect sizes.

### Three-Level Meta-Analysis

The second example was based on the data set from Stadler, Becker, Gödker, Leutner, and Greiff (2015), Table 1). These authors investigated the correlation between complex problem solving and intelligence. The authors reported the effect sizes of 60 independent samples from 47 studies. Therefore, the effect sizes were nested within the studies. In their Table 1, however, they did not provide explicit information on how these independent samples were nested. Stadler et al. (2015) conducted their meta-analysis without taking the non-independence of the effect sizes into account. Based on the information on “Authors” and “Year,” we could only identify 44 clusters. As an illustration, we conducted the three-level meta-analysis with 60 effect sizes nested within 44 studies. The number of effect sizes per study varied from 1 to 4.

If we ignore the dependence and conduct the univariate meta-analysis, the average correlation (and its *SE*) is .42 (.03). The estimated heterogeneity variance and the *I*^2^ are .04 and .96, respectively. Based on the three-level meta-analysis, the average correlation (and its *SE*) is .43 (.03). The estimated level-2 and level-3 heterogeneity variances are .02 and .02, respectively while the estimated level-2 and level-3 *I*^2^ are .45 and .51, respectively. The three-level meta-analysis provides more information on how the heterogeneity can be decomposed into the level-2 and level-3 components. The results suggest that the study level can account for more heterogeneity (51%) than the effect size level does (45%).

In the dataset, the effect sizes are based on two different intelligence measures (general intelligence, with 21 independent samples; and reasoning, with 39 independent samples). It is of interest to test whether the effects on these intelligence measures are the same. We include the intelligence measure as a moderator in the three-level meta-analysis. By comparing the models with and without the moderator, we find that the change in the chi-square statistics was *χ*^2^(*df* = 1) = 4.52, *p* = .03. The average correlation between complex problem solving and intelligence is stronger for studies with a reasoning measure, at .48 (*SE* = .04), than for those with a general intelligence measure, at .35 (*SE* = .05).

## Conclusion and Future Directions

This paper introduced the problems and preferred solutions for handling non-independent effect sizes in a meta-analysis. Multivariate meta-analyses and three-level meta-analyses can handle different types of non-independent effect sizes. Besides providing valid statistical models to handle non-independent effect sizes, multivariate and three-level meta-analyses allow researchers to address new research questions that cannot be answered in a conventional meta-analysis. In a multivariate meta-analysis, we may compare the average effects or heterogeneity variances across different types of effect sizes. We may also study how the population effect sizes are correlated. In a three-level meta-analysis, we may investigate the heterogeneity variances and explained variances at different levels.

A multivariate meta-analysis is usually more challenging to implement because we need to know the correlation between the effect sizes. Many primary studies, however, may not include information on how to estimate this correlation. In contrast, it is easier to implement a three-level meta-analysis because the degree of dependence is estimated from the data. As we have illustrated in the above example, a three-level meta-analysis may also be used to handle different types of effect sizes, namely, the outcome measure in our illustration.

In this paper, we simplify the non-independence into either multivariate or nested effect sizes. Then a multivariate meta-analysis and a three-level meta-analysis are used to address the non-independence of the effect sizes. In applied research, the type of dependence is usually more complicated than in cases with either multivariate or nested effect sizes (see, e.g., Prado, Watt, & Crowe, 2018 for an example). It may involve both multivariate outcomes and nested structures (e.g., Cheung, 2018; Scammacca, Roberts, & Stuebing, 2014). The effect sizes can be cross-classified rather than nested (Fernández-Castilla et al., 2018). Researchers may need to decide on the best models to use in analyzing the data.

The effect sizes may still be non-independent even though each study only contributes to one effect size. For example, Shin (2009) found that the effect sizes reported by the same research groups or authors tended to be more similar to each other than those reported by other research groups or authors. Moreover, the effect sizes of studies based on the same data sets are also more similar to each other. If this dependence is ignored, the estimated uncertainty (*SE*) may be biased. Ideally, we may want to model all types of dependence. However, it is sometimes challenging to do this. Further studies may clarify when it is acceptable to drop or combine the effect sizes to simplify the analyses.

Before closing this paper, it is important to discuss a few issues. First, the selection of effect sizes should be guided by the research questions. Researchers should not blindly include all effect sizes simply because the effect sizes are available. Researchers should carefully define the inclusion and exclusion criteria and use these criteria to determine whether or not the effect sizes should be included.

Another issue is the number of effect sizes needed to conduct a three-level meta-analysis. Similar to a standard meta-analysis and multilevel model, the fixed-effects estimates are usually quite stable whereas the stability of the estimated level-2 and level-3 variance components depends on the number of effect sizes for the level-2 and level-3 data. For example, López-López et al. (2017) showed that the estimated fixed effects worked very well with four effect sizes per study. Similar findings were also made in Moeyaert et al. (2017). Therefore, researchers should apply a three-level meta-analysis even when the number of level-2 effect sizes is smaller. When the number of level-2 or level-3 effect sizes is small, however, researchers should be cautious in interpreting the estimated level-2 and level-3 variance components.

In conclusion, researchers have to properly incorporate the dependence in a meta-analysis. The recent development of multivariate and three-level meta-analyses provides a good starting point from which to analyze non-independent effect sizes.

## Electronic supplementary material

ESM 1 (PDF 306 kb)
