# Supplementary material for: A Guide to Conducting a Meta-Analysis with Non-Independent Effect Sizes
Source: Neuropsychol Rev. 2019 Aug 24;29(4):387–96. doi: 10.1007/s11065-019-09415-6 (PMC6892772; doi:10.1007/s11065-019-09415-6)
Supplement: Supplementary file 1 — (PDF 306 kb) [file 11065_2019_9415_MOESM1_ESM.pdf]

Supplementary Materials for "A Guide to Conducting a Meta-Analysis with Non-Independent Effect Sizes"

Mike W.-L. Cheung

July 09, 2019

Contents

Multivariate meta-analysis with a dataset from Nam, Mengersen, and Garthwaite (2003) 1

- Preparation . . . 1
- Univariate meta-analyses . . . 2
- Multivariate meta-analysis (assuming cor=0.5 between asthma and LRD) . . . 3
- Multivariate meta-analysis (assuming cor=0 between asthma and LRD) . . . 9
- Multivariate meta-analysis (assuming cor=0.8 between asthma and LRD) . . . 14

Three-level meta-analysis with a dataset from Stadler et al. (2015) 20

- Univariate meta-analysis . . . 20
- Three-level meta-analysis . . . 21

Running the analyses in Mplus 23

- Export the data to Mplus . . . 23
- Multivariate meta-analysis . . . 24
- Three-level meta-analysis . . . 40

- A latest version of the R and Mplus code is available at https://github.com/mikewlcheung/code-in-articles.

Multivariate meta-analysis with a dataset from Nam, Mengersen, and Garthwaite (2003)

Preparation

```
## Required packages in this paper
lib2install <- c("metaSEM", "metafor")

## Install them automatically if they are not available in your computer
for (i in lib2install) {
  if (!(i %in% rownames(installed.packages()))) install.packages(i)
}

## Load the libraries
library(metaSEM)
library(metafor)

## Display the first few lines of the data
head(Nam03)
```

```
##   ID Size  Age Year
## 1  3 1285  1.1 1987
```

```
## 2  4  470  9.0 1994
## 3  6 1077  6.7 1995
## 4  8  550  1.7 1995
## 5 10  850  9.4 1996
## 6 11  892 10.9 1996
##                                                              Country
## 1                                                English or Scotland
## 2                                         Scandanavia or Netherlands
## 3                                                English or Scotland
## 4                                         Scandanavia or Netherlands
## 5 Other (Israel, Turkey, South Africa, Mexico or U. Arab Emirates
## 6                                                      USA or Canada
##               Smoke Adj Asthma_logOR  LRD_logOR Asthma_v AsthmaLRD_cov_05
## 1 Parental exposure   0   0.38526240         NA   0.0729               NA
## 2 Parental exposure   1           NA 0.03922071       NA               NA
## 3 Parental exposure   0   0.35065687         NA   0.0225               NA
## 4 Parental exposure   0           NA 0.60976557       NA               NA
## 5 Parental exposure   0   0.24686008         NA   0.0529               NA
## 6 Parental exposure   1  -0.02020271         NA   0.0484               NA
##    LRD_v
## 1     NA
## 2 0.0400
## 3     NA
## 4 0.0324
## 5     NA
## 6     NA
```

Univariate meta-analyses

```
## Univariate meta-analysis on Asthma
summary(meta(y=Asthma_logOR, v=Asthma_v, data=Nam03))
```

```
##
## Call:
## meta(y = Asthma_logOR, v = Asthma_v, data = Nam03)
##
## 95% confidence intervals: z statistic approximation (robust=FALSE)
## Coefficients:
##             Estimate Std.Error    lbound    ubound z value  Pr(>|z|)
## Intercept1 0.2323694 0.0505369 0.1333190 0.3314199  4.5980 4.265e-06 ***
## Tau2_1_1   0.0403718 0.0198923 0.0013836 0.0793600  2.0295   0.04241 *
## ---
## Signif. codes:  0 '***' 0.001 '**' 0.01 '*' 0.05 '.' 0.1 ' ' 1
##
## Q statistic on the homogeneity of effect sizes: 103.6855
## Degrees of freedom of the Q statistic: 31
## P value of the Q statistic: 9.149564e-10
##
## Heterogeneity indices (based on the estimated Tau2):
##                               Estimate
## Intercept1: I2 (Q statistic)   0.7297
##
## Number of studies (or clusters): 59
```

```
## Number of observed statistics: 32
## Number of estimated parameters: 2
## Degrees of freedom: 30
## -2 log likelihood: 16.81653
## OpenMx status1: 0 ("0" or "1": The optimization is considered fine.
## Other values may indicate problems.)
```

```
## Univariate meta-analysis on LRD
summary(meta(y=LRD_logOR, v=LRD_v, data=Nam03))
```

```
##
## Call:
## meta(y = LRD_logOR, v = LRD_v, data = Nam03)
##
## 95% confidence intervals: z statistic approximation (robust=FALSE)
## Coefficients:
##               Estimate   Std.Error      lbound      ubound z value
## Intercept1  0.29576274  0.05688763  0.18426504  0.40726045  5.1991
## Tau2_1_1    0.05081379  0.02612091 -0.00038226  0.10200984  1.9453
##             Pr(>|z|)
## Intercept1 2.003e-07 ***
## Tau2_1_1     0.05174 .
## ---
## Signif. codes:  0 '***' 0.001 '**' 0.01 '*' 0.05 '.' 0.1 ' ' 1
##
## Q statistic on the homogeneity of effect sizes: 164.6626
## Degrees of freedom of the Q statistic: 34
## P value of the Q statistic: 0
##
## Heterogeneity indices (based on the estimated Tau2):
##                               Estimate
## Intercept1: I2 (Q statistic)    0.9223
##
## Number of studies (or clusters): 59
## Number of observed statistics: 35
## Number of estimated parameters: 2
## Degrees of freedom: 33
## -2 log likelihood: 34.13923
## OpenMx status1: 0 ("0" or "1": The optimization is considered fine.
## Other values may indicate problems.)
```

Multivariate meta-analysis (assuming cor=0.5 between asthma and LRD)

Without any moderator

```
fit0a <- meta(y=cbind(Asthma_logOR, LRD_logOR),
              v=cbind(Asthma_v, AsthmaLRD_cov_05, LRD_v),
              data=Nam03,
              model.name="Multi MA")
summary(fit0a)
```

```
##
## Call:
## meta(y = cbind(Asthma_logOR, LRD_logOR), v = cbind(Asthma_v,
```

```
##     AsthmaLRD_cov_05, LRD_v), data = Nam03, model.name = "Multi MA")
##
## 95% confidence intervals: z statistic approximation (robust=FALSE)
## Coefficients:
##             Estimate Std.Error    lbound    ubound z value  Pr(>|z|)
## Intercept1 0.2681317 0.0544460 0.1614195 0.3748438  4.9247 8.448e-07 ***
## Intercept2 0.3079815 0.0541262 0.2018960 0.4140670  5.6901 1.270e-08 ***
## Tau2_1_1   0.0684487 0.0296292 0.0103766 0.1265207  2.3102  0.020878 *
## Tau2_2_1   0.0555588 0.0205440 0.0152934 0.0958243  2.7044  0.006843 **
## Tau2_2_2   0.0484633 0.0231445 0.0031009 0.0938257  2.0939  0.036265 *
## ---
## Signif. codes:  0 '***' 0.001 '**' 0.01 '*' 0.05 '.' 0.1 ' ' 1
##
## Q statistic on the homogeneity of effect sizes: 282.7301
## Degrees of freedom of the Q statistic: 65
## P value of the Q statistic: 0
##
## Heterogeneity indices (based on the estimated Tau2):
##                              Estimate
## Intercept1: I2 (Q statistic)   0.8207
## Intercept2: I2 (Q statistic)   0.9188
##
## Number of studies (or clusters): 59
## Number of observed statistics: 67
## Number of estimated parameters: 5
## Degrees of freedom: 62
## -2 log likelihood: 42.55209
## OpenMx status1: 0 ("0" or "1": The optimization is considered fine.
## Other values may indicate problems.)
```

```
## Correlation between the population effect sizes
cov2cor(VarCorr(fit0a))
```

```
##           [,1]      [,2]
## [1,] 1.0000000 0.9646376
## [2,] 0.9646376 1.0000000
```

```
## Plot the (complete) effect sizes and their 95% confidence ellipses
plot(fit0a, xlim=c(-0.5, 1), ylim=c(-0.5, 1), axis.labels=c("Asthma", "LRD"),
     study.ellipse.plot = FALSE, diag.panel=TRUE,
     randeff.ellipse.lty=2,
     main="Log-odds ratio")

forest(rma(yi=Asthma_logOR, vi=Asthma_v, slab=ID, data=Nam03),
       xlab="Log-odds ratio")
title("Forest plot of Asthma")

forest(rma(yi=LRD_logOR, vi=LRD_v, slab=ID, data=Nam03),
       xlab="Log-odds ratio")
title("Forest plot of LRD")
```

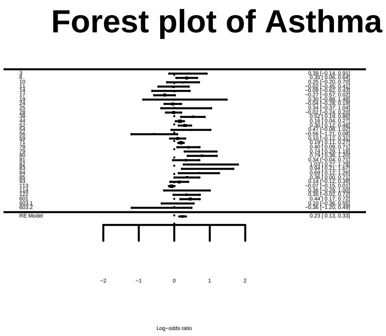

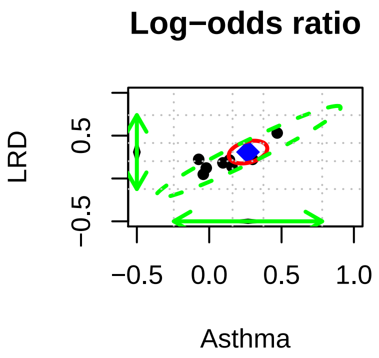

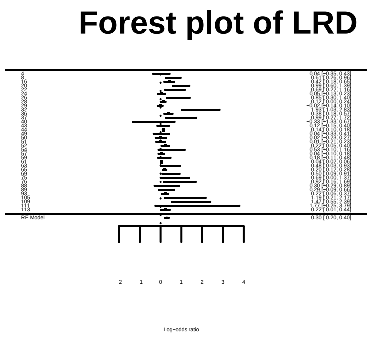


```
## Test whether (1) the population effect sizes and
## (2) heterogeneity variances are the same for Asthma and LRD
fit1a <- meta(y=cbind(Asthma_logOR, LRD_logOR),
              v=cbind(Asthma_v, AsthmaLRD_cov_05, LRD_v),
              data=Nam03,
              intercept.constraints=c("0*Intercept", "0*Intercept"),
              RE.constraints=matrix(c("0.1*Tau2_1_1", "0*Tau2_2_1",
                                      "0*Tau2_2_1", "0.1*Tau2_1_1"),
                                    ncol=2, nrow=2),
              model.name="Equality constraints")
## Compare the models with and without the equality constraints
anova(fit0a, fit1a)
```

```
##       base            comparison ep minus2LL df       AIC   diffLL diffdf
## 1 Multi MA                  <NA>  5 42.55209 62 -81.44791       NA     NA
## 2 Multi MA Equality constraints  3 45.32722 64 -82.67278 2.775128      2
##           p
## 1        NA
## 2 0.2496827
```

```
summary(fit1a)
```

```
##
## Call:
## meta(y = cbind(Asthma_logOR, LRD_logOR), v = cbind(Asthma_v,
##     AsthmaLRD_cov_05, LRD_v), data = Nam03, intercept.constraints = c("0*Intercept",
##     "0*Intercept"), RE.constraints = matrix(c("0.1*Tau2_1_1",
```

```
##     "0*Tau2_2_1", "0*Tau2_2_1", "0.1*Tau2_1_1"), ncol = 2, nrow = 2),
##     model.name = "Equality constraints")
##
## 95% confidence intervals: z statistic approximation (robust=FALSE)
## Coefficients:
##            Estimate Std.Error    lbound    ubound z value  Pr(>|z|)
## Intercept 0.2927135 0.0429388 0.2085549 0.3768721  6.8170 9.297e-12 ***
## Tau2_1_1  0.0551239 0.0199668 0.0159896 0.0942582  2.7608  0.005766 **
## Tau2_2_1  0.0497245 0.0209576 0.0086484 0.0908005  2.3726  0.017662 *
## ---
## Signif. codes:  0 '***' 0.001 '**' 0.01 '*' 0.05 '.' 0.1 ' ' 1
##
## Q statistic on the homogeneity of effect sizes: 282.7301
## Degrees of freedom of the Q statistic: 65
## P value of the Q statistic: 0
##
## Heterogeneity indices (based on the estimated Tau2):
##                              Estimate
## Intercept1: I2 (Q statistic)   0.7866
## Intercept2: I2 (Q statistic)   0.9279
##
## Number of studies (or clusters): 59
## Number of observed statistics: 67
## Number of estimated parameters: 3
## Degrees of freedom: 64
## -2 log likelihood: 45.32722
## OpenMx status1: 0 ("0" or "1": The optimization is considered fine.
## Other values may indicate problems.)
```

With the mean Age as a moderator

```
fit2a <- meta(y=cbind(Asthma_logOR, LRD_logOR),
              v=cbind(Asthma_v, AsthmaLRD_cov_05, LRD_v),
              ## Center Age for ease of interpretations
              x=scale(Age, scale=FALSE),
              ## Fix the covariance of between the random effects at 0
              ## as there is not enough data
              RE.constraints=matrix(c("0.1*Tau2_1_1", "0",
                                      "0", "0.1*Tau2_2_2"),
                                    ncol=2, nrow=2),
              data=Nam03,
              model.name="Mixed MA")
summary(fit2a)
```

```
##
## Call:
## meta(y = cbind(Asthma_logOR, LRD_logOR), v = cbind(Asthma_v,
##     AsthmaLRD_cov_05, LRD_v), x = scale(Age, scale = FALSE),
##     data = Nam03, RE.constraints = matrix(c("0.1*Tau2_1_1", "0",
##         "0", "0.1*Tau2_2_2"), ncol = 2, nrow = 2), model.name = "Mixed MA")
##
## 95% confidence intervals: z statistic approximation (robust=FALSE)
## Coefficients:
```

```
##                Estimate   Std.Error      lbound      ubound z value
## Intercept1  0.26807390  0.04865798  0.17270602  0.36344178  5.5094
## Intercept2  0.27292919  0.04744385  0.17994095  0.36591742  5.7527
## Slope1_1   -0.03809070  0.01558287 -0.06863257 -0.00754882 -2.4444
## Slope2_1   -0.02366521  0.00935199 -0.04199477 -0.00533565 -2.5305
## Tau2_1_1    0.02831617  0.01439380  0.00010483  0.05652751  1.9672
## Tau2_2_2    0.02970105  0.01560339 -0.00088104  0.06028314  1.9035
##              Pr(>|z|)
## Intercept1 3.602e-08 ***
## Intercept2 8.784e-09 ***
## Slope1_1     0.01451 *
## Slope2_1     0.01139 *
## Tau2_1_1     0.04915 *
## Tau2_2_2     0.05698 .
## ---
## Signif. codes:  0 '***' 0.001 '**' 0.01 '*' 0.05 '.' 0.1 ' ' 1
##
## Q statistic on the homogeneity of effect sizes: 282.7301
## Degrees of freedom of the Q statistic: 65
## P value of the Q statistic: 0
##
## Explained variances (R2):
##                               y1     y2
## Tau2 (no predictor)    0.068449 0.0485
## Tau2 (with predictors) 0.028316 0.0297
## R2                     0.586315 0.3871
##
## Number of studies (or clusters): 59
## Number of observed statistics: 67
## Number of estimated parameters: 6
## Degrees of freedom: 61
## -2 log likelihood: 38.83728
## OpenMx status1: 0 ("0" or "1": The optimization is considered fine.
## Other values may indicate problems.)
```

```
## Test whether the effect of mean Age the same for Asthma and LRD
fit3a <- meta(y=cbind(Asthma_logOR, LRD_logOR),
              v=cbind(Asthma_v, AsthmaLRD_cov_05, LRD_v),
              ## Center Age for ease of interpretations
              x=scale(Age, scale=FALSE),
              ## Fix the covariance of between the random effects at 0
              ## as there is not enough data
              RE.constraints=matrix(c("0.1*Tau2_1_1", "0",
                                      "0", "0.1*Tau2_2_2"),
                                    ncol=2, nrow=2),
              ## Fix the regression coefficients to be identical by
              ## using the same label `Slope1`.
              coef.constraints=matrix(matrix(c("0*Slope1", "0*Slope1"),
                                             nrow=1)),
              data=Nam03,
              model.name="Equality constraints")

## Compare the models with and without the equality constraints
anova(fit2a, fit3a)
```

```
##       base           comparison ep minus2LL df       AIC    diffLL diffdf
## 1 Mixed MA                 <NA>  6 38.83728 61 -83.16272        NA     NA
## 2 Mixed MA Equality constraints  5 39.47544 62 -84.52456 0.6381537      1
##           p
## 1        NA
## 2 0.4243802
```

```r
summary(fit3a)
```

```
##
## Call:
## meta(y = cbind(Asthma_logOR, LRD_logOR), v = cbind(Asthma_v,
##     AsthmaLRD_cov_05, LRD_v), x = scale(Age, scale = FALSE),
##     data = Nam03, coef.constraints = matrix(matrix(c("0*Slope1",
##         "0*Slope1"), nrow = 1)), RE.constraints = matrix(c("0.1*Tau2_1_1",
##         "0", "0", "0.1*Tau2_2_2"), ncol = 2, nrow = 2), model.name = "Equality constraints")
##
## 95% confidence intervals: z statistic approximation (robust=FALSE)
## Coefficients:
##               Estimate   Std.Error      lbound      ubound z value
## Intercept1  0.25595987  0.04598891  0.16582326  0.34609648  5.5657
## Intercept2  0.27564678  0.04748103  0.18258568  0.36870787  5.8054
## Slope1     -0.02760823  0.00826731 -0.04381186 -0.01140460 -3.3394
## Tau2_1_1    0.02808574  0.01465925 -0.00064586  0.05681735  1.9159
## Tau2_2_2    0.03111654  0.01582491  0.00010029  0.06213278  1.9663
##             Pr(>|z|)
## Intercept1 2.611e-08 ***
## Intercept2 6.421e-09 ***
## Slope1     0.0008395 ***
## Tau2_1_1   0.0553771 .
## Tau2_2_2   0.0492638 *
## ---
## Signif. codes:  0 '***' 0.001 '**' 0.01 '*' 0.05 '.' 0.1 ' ' 1
##
## Q statistic on the homogeneity of effect sizes: 282.7301
## Degrees of freedom of the Q statistic: 65
## P value of the Q statistic: 0
##
## Explained variances (R2):
##                               y1     y2
## Tau2 (no predictor)    0.068449 0.0485
## Tau2 (with predictors) 0.028086 0.0311
## R2                     0.589682 0.3579
##
## Number of studies (or clusters): 59
## Number of observed statistics: 67
## Number of estimated parameters: 5
## Degrees of freedom: 62
## -2 log likelihood: 39.47544
## OpenMx status1: 0 ("0" or "1": The optimization is considered fine.
## Other values may indicate problems.)
```

Multivariate meta-analysis (assuming cor=0 between asthma and LRD)

Without any moderator

```
## Create a variable of sampling correlation=0
Nam03$AsthmaLRD_cov_0 <- 0

fit0b <- meta(y=cbind(Asthma_logOR, LRD_logOR),
              v=cbind(Asthma_v, AsthmaLRD_cov_0, LRD_v),
              data=Nam03,
              model.name="Multi MA")
summary(fit0b)
```

```
##
## Call:
## meta(y = cbind(Asthma_logOR, LRD_logOR), v = cbind(Asthma_v,
##     AsthmaLRD_cov_0, LRD_v), data = Nam03, model.name = "Multi MA")
##
## 95% confidence intervals: z statistic approximation (robust=FALSE)
## Coefficients:
##             Estimate Std.Error    lbound    ubound z value  Pr(>|z|)
## Intercept1 0.2659166 0.0544499 0.1591968 0.3726363  4.8837 1.041e-06 ***
## Intercept2 0.3135788 0.0563451 0.2031444 0.4240132  5.5653 2.617e-08 ***
## Tau2_1_1   0.0635368 0.0279824 0.0086923 0.1183814  2.2706  0.023171 *
## Tau2_2_1   0.0560841 0.0205646 0.0157782 0.0963901  2.7272  0.006387 **
## Tau2_2_2   0.0521575 0.0247765 0.0035964 0.1007185  2.1051  0.035281 *
## ---
## Signif. codes:  0 '***' 0.001 '**' 0.01 '*' 0.05 '.' 0.1 ' ' 1
##
## Q statistic on the homogeneity of effect sizes: 268.3481
## Degrees of freedom of the Q statistic: 65
## P value of the Q statistic: 0
##
## Heterogeneity indices (based on the estimated Tau2):
##                               Estimate
## Intercept1: I2 (Q statistic)    0.8095
## Intercept2: I2 (Q statistic)    0.9241
##
## Number of studies (or clusters): 59
## Number of observed statistics: 67
## Number of estimated parameters: 5
## Degrees of freedom: 62
## -2 log likelihood: 44.17176
## OpenMx status1: 0 ("0" or "1": The optimization is considered fine.
## Other values may indicate problems.)
```

```
## Correlation between the population effect sizes
cov2cor(VarCorr(fit0b))
```

```
##          [,1]     [,2]
## [1,] 1.000000 0.974247
## [2,] 0.974247 1.000000
```

```
## Plot the (complete) effect sizes and their 95% confidence ellipses
plot(fit0b, xlim=c(-0.5, 1), ylim=c(-0.5, 1), axis.labels=c("Asthma", "LRD"),
```

```r
      study.ellipse.plot = FALSE, diag.panel=TRUE,
      randeff.ellipse.lty=2,
      main="Log-odds ratio (assuming Cor=0)")

forest(rma(yi=Asthma_logOR, vi=Asthma_v, slab=ID, data=Nam03),
       xlab="Log-odds ratio")
```

```
## Warning in rma(yi = Asthma_logOR, vi = Asthma_v, slab = ID, data = Nam03):
## Studies with NAs omitted from model fitting.
```

```r
title("Forest plot of Asthma")

forest(rma(yi=LRD_logOR, vi=LRD_v, slab=ID, data=Nam03),
       xlab="Log-odds ratio")
```

```
## Warning in rma(yi = LRD_logOR, vi = LRD_v, slab = ID, data = Nam03):
## Studies with NAs omitted from model fitting.
```

```r
title("Forest plot of LRD")
```

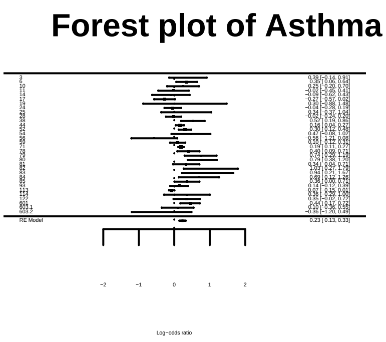

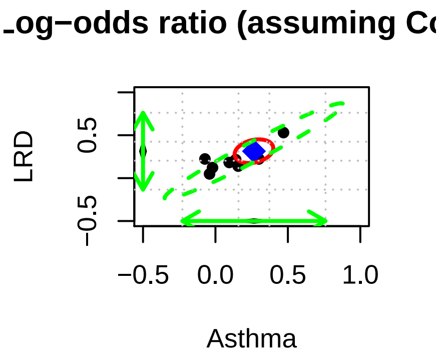

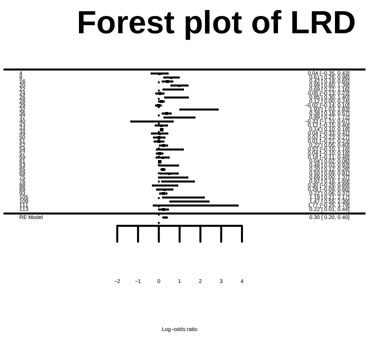


```r
## Test whether (1) the population effect sizes and
## (2) heterogeneity variances are the same for Asthma and LRD
fit1b <- meta(y=cbind(Asthma_logOR, LRD_logOR),
              v=cbind(Asthma_v, AsthmaLRD_cov_0, LRD_v),
              data=Nam03,
              intercept.constraints=c("0*Intercept", "0*Intercept"),
              RE.constraints=matrix(c("0.1*Tau2_1_1", "0*Tau2_2_1",
```

```r
                                       "0*Tau2_2_1", "0.1*Tau2_1_1"),
                                     ncol=2, nrow=2),
              model.name="Equality constraints")
## Compare the models with and without the equality constraints
anova(fit0b, fit1b)
```

```
##       base           comparison ep minus2LL df       AIC   diffLL diffdf
## 1 Multi MA                 <NA>  5 44.17176 62 -79.82824       NA     NA
## 2 Multi MA Equality constraints  3 46.29695 64 -81.70305 2.125192      2
##           p
## 1        NA
## 2 0.3455577
```

```r
summary(fit1b)
```

```
##
## Call:
## meta(y = cbind(Asthma_logOR, LRD_logOR), v = cbind(Asthma_v,
##     AsthmaLRD_cov_0, LRD_v), data = Nam03, intercept.constraints = c("0*Intercept",
##     "0*Intercept"), RE.constraints = matrix(c("0.1*Tau2_1_1",
##     "0*Tau2_2_1", "0*Tau2_2_1", "0.1*Tau2_1_1"), ncol = 2, nrow = 2),
##     model.name = "Equality constraints")
##
## 95% confidence intervals: z statistic approximation (robust=FALSE)
## Coefficients:
##           Estimate Std.Error   lbound   ubound z value  Pr(>|z|)
## Intercept 0.292847  0.042852 0.208859 0.376835  6.8339 8.262e-12 ***
## Tau2_1_1  0.055253  0.019911 0.016228 0.094279  2.7750  0.005521 **
## Tau2_2_1  0.052059  0.021166 0.010574 0.093544  2.4596  0.013911 *
## ---
## Signif. codes:  0 '***' 0.001 '**' 0.01 '*' 0.05 '.' 0.1 ' ' 1
##
## Q statistic on the homogeneity of effect sizes: 268.3481
## Degrees of freedom of the Q statistic: 65
## P value of the Q statistic: 0
##
## Heterogeneity indices (based on the estimated Tau2):
##                              Estimate
## Intercept1: I2 (Q statistic)   0.7870
## Intercept2: I2 (Q statistic)   0.9281
##
## Number of studies (or clusters): 59
## Number of observed statistics: 67
## Number of estimated parameters: 3
## Degrees of freedom: 64
## -2 log likelihood: 46.29695
## OpenMx status1: 0 ("0" or "1": The optimization is considered fine.
## Other values may indicate problems.)
```

With the mean Age as a moderator

```r
fit2b <- meta(y=cbind(Asthma_logOR, LRD_logOR),
              v=cbind(Asthma_v, AsthmaLRD_cov_0, LRD_v),
```

```
            ## Center Age for ease of interpretations
            x=scale(Age, scale=FALSE),
            ## Fix the covariance of between the random effects at 0
            ## as there is not enough data
            RE.constraints=matrix(c("0.1*Tau2_1_1", "0",
                                    "0", "0.1*Tau2_2_2"),
                                  ncol=2, nrow=2),
            data=Nam03,
            model.name="Mixed MA")
summary(fit2b)
```

```
##
## Call:
## meta(y = cbind(Asthma_logOR, LRD_logOR), v = cbind(Asthma_v,
##     AsthmaLRD_cov_0, LRD_v), x = scale(Age, scale = FALSE), data = Nam03,
##     RE.constraints = matrix(c("0.1*Tau2_1_1", "0", "0", "0.1*Tau2_2_2"),
##         ncol = 2, nrow = 2), model.name = "Mixed MA")
##
## 95% confidence intervals: z statistic approximation (robust=FALSE)
## Coefficients:
##                Estimate   Std.Error      lbound      ubound z value
## Intercept1  0.26839859  0.04968285  0.17102199  0.36577520  5.4022
## Intercept2  0.27343978  0.04870472  0.17798029  0.36889927  5.6142
## Slope1_1   -0.03802625  0.01589945 -0.06918861 -0.00686390 -2.3917
## Slope2_1   -0.02375587  0.00956054 -0.04249418 -0.00501756 -2.4848
## Tau2_1_1    0.03037997  0.01525122  0.00048812  0.06027182  1.9920
## Tau2_2_2    0.03189970  0.01675576 -0.00094098  0.06474039  1.9038
##             Pr(>|z|)
## Intercept1 6.581e-08 ***
## Intercept2 1.974e-08 ***
## Slope1_1     0.01677 *
## Slope2_1     0.01296 *
## Tau2_1_1     0.04637 *
## Tau2_2_2     0.05694 .
## ---
## Signif. codes:  0 '***' 0.001 '**' 0.01 '*' 0.05 '.' 0.1 ' ' 1
##
## Q statistic on the homogeneity of effect sizes: 268.3481
## Degrees of freedom of the Q statistic: 65
## P value of the Q statistic: 0
##
## Explained variances (R2):
##                                 y1     y2
## Tau2 (no predictor)    0.063537 0.0522
## Tau2 (with predictors) 0.030380 0.0319
## R2                     0.521853 0.3884
##
## Number of studies (or clusters): 59
## Number of observed statistics: 67
## Number of estimated parameters: 6
## Degrees of freedom: 61
## -2 log likelihood: 39.97773
## OpenMx status1: 0 ("0" or "1": The optimization is considered fine.
## Other values may indicate problems.)
```

```r
## Test whether the effect of mean Age the same for Asthma and LRD
fit3b <- meta(y=cbind(Asthma_logOR, LRD_logOR),
              v=cbind(Asthma_v, AsthmaLRD_cov_0, LRD_v),
              ## Center Age for ease of interpretations
              x=scale(Age, scale=FALSE),
              ## Fix the covariance of between the random effects at 0
              ## as there is not enough data
              RE.constraints=matrix(c("0.1*Tau2_1_1", "0",
                                      "0", "0.1*Tau2_2_2"),
                                    ncol=2, nrow=2),
              ## Fix the regression coefficients to be identical by
              ## using the same label `Slope1`.
              coef.constraints=matrix(matrix(c("0*Slope1", "0*Slope1"),
                                             nrow=1)),
              data=Nam03,
              model.name="Equality constraints")

## Compare the models with and without the equality constraints
anova(fit2b, fit3b)
```

```
##       base           comparison ep minus2LL df       AIC   diffLL diffdf
## 1 Mixed MA                 <NA>  6 39.97773 61 -82.02227       NA     NA
## 2 Mixed MA Equality constraints  5 40.57193 62 -83.42807 0.594199      1
##        p
## 1     NA
## 2 0.4408
```

```r
summary(fit3b)
```

```
##
## Call:
## meta(y = cbind(Asthma_logOR, LRD_logOR), v = cbind(Asthma_v,
##     AsthmaLRD_cov_0, LRD_v), x = scale(Age, scale = FALSE), data = Nam03,
##     coef.constraints = matrix(matrix(c("0*Slope1", "0*Slope1"),
##         nrow = 1)), RE.constraints = matrix(c("0.1*Tau2_1_1",
##         "0", "0", "0.1*Tau2_2_2"), ncol = 2, nrow = 2), model.name = "Equality constraints")
##
## 95% confidence intervals: z statistic approximation (robust=FALSE)
## Coefficients:
##               Estimate   Std.Error      lbound      ubound z value
## Intercept1  2.5673e-01  4.7057e-02  1.6450e-01  3.4896e-01  5.4558
## Intercept2  2.7559e-01  4.8693e-02  1.8015e-01  3.7103e-01  5.6597
## Slope1     -2.7675e-02  8.3864e-03 -4.4112e-02 -1.1238e-02 -3.3000
## Tau2_1_1    3.0141e-02  1.5511e-02 -2.5987e-04  6.0542e-02  1.9432
## Tau2_2_2    3.3103e-02  1.6914e-02 -4.7814e-05  6.6255e-02  1.9571
##              Pr(>|z|)
## Intercept1 4.876e-08 ***
## Intercept2 1.517e-08 ***
## Slope1     0.0009668 ***
## Tau2_1_1   0.0519908 .
## Tau2_2_2   0.0503313 .
## ---
## Signif. codes:  0 '***' 0.001 '**' 0.01 '*' 0.05 '.' 0.1 ' ' 1
##
```

```
## Q statistic on the homogeneity of effect sizes: 268.3481
## Degrees of freedom of the Q statistic: 65
## P value of the Q statistic: 0
##
## Explained variances (R2):
##                               y1     y2
## Tau2 (no predictor)    0.063537 0.0522
## Tau2 (with predictors) 0.030141 0.0331
## R2                     0.525614 0.3653
##
## Number of studies (or clusters): 59
## Number of observed statistics: 67
## Number of estimated parameters: 5
## Degrees of freedom: 62
## -2 log likelihood: 40.57193
## OpenMx status1: 0 ("0" or "1": The optimization is considered fine.
## Other values may indicate problems.)
```

Multivariate meta-analysis (assuming cor=0.8 between asthma and LRD)

Without any moderator

```
## Create a variable of sampling correlation=0
Nam03$AsthmaLRD_cov_08 <- with(Nam03, 0.8*sqrt(Asthma_v)*sqrt(LRD_v))

fit0c <- meta(y=cbind(Asthma_logOR, LRD_logOR),
              v=cbind(Asthma_v, AsthmaLRD_cov_08, LRD_v),
              data=Nam03,
              model.name="Multi MA")
summary(fit0c)
```

```
##
## Call:
## meta(y = cbind(Asthma_logOR, LRD_logOR), v = cbind(Asthma_v,
##     AsthmaLRD_cov_08, LRD_v), data = Nam03, model.name = "Multi MA")
##
## 95% confidence intervals: z statistic approximation (robust=FALSE)
## Coefficients:
##            Estimate Std.Error   lbound   ubound z value  Pr(>|z|)
## Intercept1 0.271590  0.054237 0.165288 0.377892  5.0075 5.515e-07 ***
## Intercept2 0.300680  0.051778 0.199198 0.402163  5.8071 6.355e-09 ***
## Tau2_1_1   0.073193  0.030757 0.012911 0.133475  2.3798  0.017324 *
## Tau2_2_1   0.055179  0.020340 0.015313 0.095044  2.7128  0.006671 **
## Tau2_2_2   0.044732  0.021433 0.002724 0.086741  2.0871  0.036883 *
## ---
## Signif. codes:  0 '***' 0.001 '**' 0.01 '*' 0.05 '.' 0.1 ' ' 1
##
## Q statistic on the homogeneity of effect sizes: 318.3938
## Degrees of freedom of the Q statistic: 65
## P value of the Q statistic: 0
##
## Heterogeneity indices (based on the estimated Tau2):
##                               Estimate
```

```
## Intercept1: I2 (Q statistic)   0.8303
## Intercept2: I2 (Q statistic)   0.9126
##
## Number of studies (or clusters): 59
## Number of observed statistics: 67
## Number of estimated parameters: 5
## Degrees of freedom: 62
## -2 log likelihood: 40.83324
## OpenMx status1: 0 ("0" or "1": The optimization is considered fine.
## Other values may indicate problems.)
```

```
## Correlation between the population effect sizes
cov2cor(VarCorr(fit0c))
```

```
##          [,1]     [,2]
## [1,] 1.000000 0.964331
## [2,] 0.964331 1.000000
```

```
## Plot the (complete) effect sizes and their 95% confidence ellipses
plot(fit0c, xlim=c(-0.5, 1), ylim=c(-0.5, 1), axis.labels=c("Asthma", "LRD"),
     study.ellipse.plot = FALSE, diag.panel=TRUE,
     randeff.ellipse.lty=2,
     main="Log-odds ratio (assuming Cor=0.8)")

forest(rma(yi=Asthma_logOR, vi=Asthma_v, slab=ID, data=Nam03),
       xlab="Log-odds ratio")
```

```
## Warning in rma(yi = Asthma_logOR, vi = Asthma_v, slab = ID, data = Nam03):
## Studies with NAs omitted from model fitting.
```

```
title("Forest plot of Asthma")

forest(rma(yi=LRD_logOR, vi=LRD_v, slab=ID, data=Nam03),
       xlab="Log-odds ratio")
```

```
## Warning in rma(yi = LRD_logOR, vi = LRD_v, slab = ID, data = Nam03):
## Studies with NAs omitted from model fitting.
```

```
title("Forest plot of LRD")
```

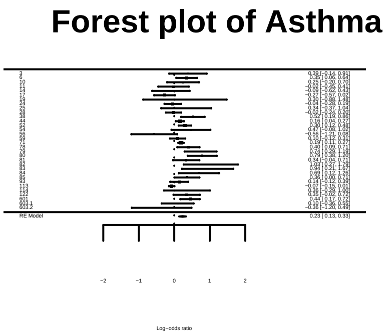

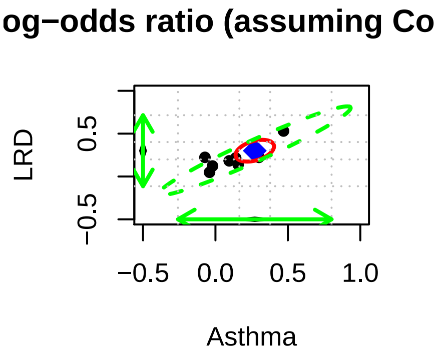

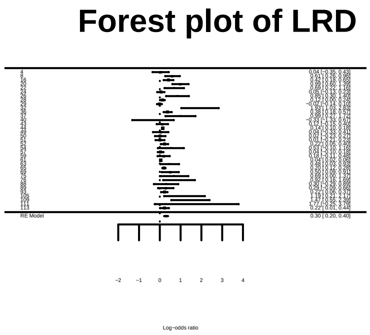


```
## Test whether (1) the population effect sizes and
## (2) heterogeneity variances are the same for Asthma and LRD
fit1c <- meta(y=cbind(Asthma_logOR, LRD_logOR),
              v=cbind(Asthma_v, AsthmaLRD_cov_08, LRD_v),
              data=Nam03,
              intercept.constraints=c("0*Intercept", "0*Intercept"),
              RE.constraints=matrix(c("0.1*Tau2_1_1", "0*Tau2_2_1",
                                      "0*Tau2_2_1", "0.1*Tau2_1_1"),
                                    ncol=2, nrow=2),
              model.name="Equality constraints")
## Compare the models with and without the equality constraints
anova(fit0c, fit1c)
```

```
##       base           comparison ep minus2LL df       AIC   diffLL diffdf
## 1 Multi MA                 <NA>  5 40.83324 62 -83.16676       NA     NA
## 2 Multi MA Equality constraints  3 44.31864 64 -83.68136 3.485405      2
##           p
## 1        NA
## 2 0.1750467
```

```
summary(fit1c)
```

```
##
## Call:
## meta(y = cbind(Asthma_logOR, LRD_logOR), v = cbind(Asthma_v,
##     AsthmaLRD_cov_08, LRD_v), data = Nam03, intercept.constraints = c("0*Intercept",
##     "0*Intercept"), RE.constraints = matrix(c("0.1*Tau2_1_1",
```

```
##     "0*Tau2_2_1", "0*Tau2_2_1", "0.1*Tau2_1_1"), ncol = 2, nrow = 2),
##     model.name = "Equality constraints")
##
## 95% confidence intervals: z statistic approximation (robust=FALSE)
## Coefficients:
##            Estimate Std.Error    lbound    ubound z value  Pr(>|z|)
## Intercept 0.2927176 0.0429686 0.2085008 0.3769344  6.8124 9.601e-12 ***
## Tau2_1_1  0.0550842 0.0199933 0.0158981 0.0942703  2.7551  0.005867 **
## Tau2_2_1  0.0486815 0.0207999 0.0079144 0.0894486  2.3405  0.019260 *
## ---
## Signif. codes:  0 '***' 0.001 '**' 0.01 '*' 0.05 '.' 0.1 ' ' 1
##
## Q statistic on the homogeneity of effect sizes: 318.3938
## Degrees of freedom of the Q statistic: 65
## P value of the Q statistic: 0
##
## Heterogeneity indices (based on the estimated Tau2):
##                               Estimate
## Intercept1: I2 (Q statistic)    0.7865
## Intercept2: I2 (Q statistic)    0.9279
##
## Number of studies (or clusters): 59
## Number of observed statistics: 67
## Number of estimated parameters: 3
## Degrees of freedom: 64
## -2 log likelihood: 44.31864
## OpenMx status1: 0 ("0" or "1": The optimization is considered fine.
## Other values may indicate problems.)
```

With the mean Age as a moderator

```
fit2c <- meta(y=cbind(Asthma_logOR, LRD_logOR),
              v=cbind(Asthma_v, AsthmaLRD_cov_08, LRD_v),
              ## Center Age for ease of interpretations
              x=scale(Age, scale=FALSE),
              ## Fix the covariance of between the random effects at 0
              ## as there is not enough data
              RE.constraints=matrix(c("0.1*Tau2_1_1", "0",
                                      "0", "0.1*Tau2_2_2"),
                                    ncol=2, nrow=2),
              data=Nam03,
              model.name="Mixed MA")
summary(fit2c)
```

```
##
## Call:
## meta(y = cbind(Asthma_logOR, LRD_logOR), v = cbind(Asthma_v,
##     AsthmaLRD_cov_08, LRD_v), x = scale(Age, scale = FALSE),
##     data = Nam03, RE.constraints = matrix(c("0.1*Tau2_1_1", "0",
##         "0", "0.1*Tau2_2_2"), ncol = 2, nrow = 2), model.name = "Mixed MA")
##
## 95% confidence intervals: z statistic approximation (robust=FALSE)
## Coefficients:
```

```
##                Estimate   Std.Error      lbound      ubound z value
## Intercept1  2.6742e-01  4.7779e-02  1.7378e-01  3.6107e-01  5.5970
## Intercept2  2.7264e-01  4.6412e-02  1.8168e-01  3.6361e-01  5.8744
## Slope1_1   -3.8071e-02  1.5347e-02 -6.8151e-02 -7.9907e-03 -2.4806
## Slope2_1   -2.3580e-02  9.2084e-03 -4.1628e-02 -5.5316e-03 -2.5607
## Tau2_1_1    2.6934e-02  1.3733e-02  1.7209e-05  5.3850e-02  1.9612
## Tau2_2_2    2.8248e-02  1.4782e-02 -7.2416e-04  5.7220e-02  1.9110
##              Pr(>|z|)
## Intercept1 2.181e-08 ***
## Intercept2 4.243e-09 ***
## Slope1_1     0.01312 *
## Slope2_1     0.01045 *
## Tau2_1_1     0.04985 *
## Tau2_2_2     0.05601 .
## ---
## Signif. codes:  0 '***' 0.001 '**' 0.01 '*' 0.05 '.' 0.1 ' ' 1
##
## Q statistic on the homogeneity of effect sizes: 318.3938
## Degrees of freedom of the Q statistic: 65
## P value of the Q statistic: 0
##
## Explained variances (R2):
##                              y1     y2
## Tau2 (no predictor)    0.073193 0.0447
## Tau2 (with predictors) 0.026934 0.0282
## R2                     0.632020 0.3685
##
## Number of studies (or clusters): 59
## Number of observed statistics: 67
## Number of estimated parameters: 6
## Degrees of freedom: 61
## -2 log likelihood: 37.96009
## OpenMx status1: 0 ("0" or "1": The optimization is considered fine.
## Other values may indicate problems.)
```

```
## Test whether the effect of mean Age the same for Asthma and LRD
fit3c <- meta(y=cbind(Asthma_logOR, LRD_logOR),
              v=cbind(Asthma_v, AsthmaLRD_cov_08, LRD_v),
              ## Center Age for ease of interpretations
              x=scale(Age, scale=FALSE),
              ## Fix the covariance of between the random effects at 0
              ## as there is not enough data
              RE.constraints=matrix(c("0.1*Tau2_1_1", "0",
                                      "0", "0.1*Tau2_2_2"),
                                    ncol=2, nrow=2),
              ## Fix the regression coefficients to be identical by
              ## using the same label `Slope1`.
              coef.constraints=matrix(matrix(c("0*Slope1", "0*Slope1"),
                                             nrow=1)),
              data=Nam03,
              model.name="Equality constraints")

## Compare the models with and without the equality constraints
anova(fit2c, fit3c)
```

```
##       base           comparison ep minus2LL df       AIC    diffLL diffdf
## 1 Mixed MA                 <NA>  6 37.96009 61 -84.03991        NA     NA
## 2 Mixed MA Equality constraints  5 38.62837 62 -85.37163 0.6682878      1
##           p
## 1        NA
## 2 0.4136492
```

```
summary(fit3c)
```

```
##
## Call:
## meta(y = cbind(Asthma_logOR, LRD_logOR), v = cbind(Asthma_v,
##     AsthmaLRD_cov_08, LRD_v), x = scale(Age, scale = FALSE),
##     data = Nam03, coef.constraints = matrix(matrix(c("0*Slope1",
##         "0*Slope1"), nrow = 1)), RE.constraints = matrix(c("0.1*Tau2_1_1",
##         "0", "0", "0.1*Tau2_2_2"), ncol = 2, nrow = 2), model.name = "Equality constraints")
##
## 95% confidence intervals: z statistic approximation (robust=FALSE)
## Coefficients:
##               Estimate   Std.Error      lbound      ubound z value
## Intercept1  0.25511009  0.04511419  0.16668790  0.34353228  5.6548
## Intercept2  0.27567384  0.04648069  0.18457336  0.36677432  5.9309
## Slope1     -0.02753187  0.00818095 -0.04356623 -0.01149750 -3.3654
## Tau2_1_1    0.02670746  0.01399698 -0.00072612  0.05414104  1.9081
## Tau2_2_2    0.02976481  0.01503751  0.00029183  0.05923779  1.9794
##             Pr(>|z|)
## Intercept1 1.561e-08 ***
## Intercept2 3.012e-09 ***
## Slope1     0.0007644 ***
## Tau2_1_1   0.0563800 .
## Tau2_2_2   0.0477743 *
## ---
## Signif. codes:  0 '***' 0.001 '**' 0.01 '*' 0.05 '.' 0.1 ' ' 1
##
## Q statistic on the homogeneity of effect sizes: 318.3938
## Degrees of freedom of the Q statistic: 65
## P value of the Q statistic: 0
##
## Explained variances (R2):
##                              y1     y2
## Tau2 (no predictor)    0.073193 0.0447
## Tau2 (with predictors) 0.026707 0.0298
## R2                     0.635109 0.3346
##
## Number of studies (or clusters): 59
## Number of observed statistics: 67
## Number of estimated parameters: 5
## Degrees of freedom: 62
## -2 log likelihood: 38.62837
## OpenMx status1: 0 ("0" or "1": The optimization is considered fine.
## Other values may indicate problems.)
```

Three-level meta-analysis with a dataset from Stadler et al. (2015)

Univariate meta-analysis

```r
## Show the first few cases
head(Stadler15)
```

```
##   ID                 Authors Year   N CPSMeasure  IntelligenceMeasure    r
## 1  1      Abele et al. (2012) 2012 167        MCS            Reasoning 0.40
## 2  2 Beckman & Guthke (1995) 1995  92  Classical General intelligence 0.15
## 3  3     Buhner et al. (2008) 2008 144        SCS            Reasoning 0.16
## 4  4 Burkolter et al. (2009) 2009  41  Classical General intelligence 0.75
## 5  5 Burkolter et al. (2010) 2010  39  Classical General intelligence 0.22
## 6  6        Burmeister (2009) 2009  44  Classical General intelligence 0.47
##             v
## 1 0.004250602
## 2 0.010500069
## 3 0.006639548
## 4 0.004785156
## 5 0.023830067
## 6 0.014116205
```

```r
summary(meta(y=r, v=v, data=Stadler15))
```

```
##
## Call:
## meta(y = r, v = v, data = Stadler15)
##
## 95% confidence intervals: z statistic approximation (robust=FALSE)
## Coefficients:
##             Estimate Std.Error    lbound    ubound z value  Pr(>|z|)
## Intercept1 0.4209997 0.0304717 0.3612762 0.4807232 13.8161 < 2.2e-16 ***
## Tau2_1_1   0.0423946 0.0097469 0.0232911 0.0614982  4.3496 1.364e-05 ***
## ---
## Signif. codes:  0 '***' 0.001 '**' 0.01 '*' 0.05 '.' 0.1 ' ' 1
##
## Q statistic on the homogeneity of effect sizes: 1155.141
## Degrees of freedom of the Q statistic: 59
## P value of the Q statistic: 0
##
## Heterogeneity indices (based on the estimated Tau2):
##                               Estimate
## Intercept1: I2 (Q statistic)    0.9624
##
## Number of studies (or clusters): 60
## Number of observed statistics: 60
## Number of estimated parameters: 2
## Degrees of freedom: 58
## -2 log likelihood: -2.06745
## OpenMx status1: 0 ("0" or "1": The optimization is considered fine.
## Other values may indicate problems.)
```

Three-level meta-analysis

Without any moderator

```
## Display the number of effect sizes per study
table(Stadler15$Authors)
```

```
##
##               Abele et al. (2012)          Beckman & Guthke (1995)
##                                 1                                1
##              Buhner et al. (2008)          Burkolter et al. (2009)
##                                 1                                1
##           Burkolter et al. (2010)                Burmeister (2009)
##                                 1                                1
##                    Danner (2011)             Dorner et al. (1983)
##                                 1                                2
##              Gediga et al. (1984)           Gonzales et al. (2005)
##                                 1                                3
##           Greiff & Fischer (2013)             Greiff et al. (2015)
##                                 1                                2
##              Guss & Dorner (2011)                Hasselmann (1993)
##                                 1                                1
##                     Hesse (1982)          Hormann & Thomas (1989)
##                                 4                                2
##                     Hussy (1985)                     Hussy (1989)
##                                 4                                1
##                  Kersting (2001)             Klieme et al. (2001)
##                                 1                                1
##               Kluge et al. (2011)              Kretzschmar (2010)
##                                 1                                1
## Kretzschmar et al. (Unpublished)                    Kroner (2001)
##                                 1                                1
##              Kroner et al. (2005)                   Leutner (2002)
##                                 1                                2
##             Leutner et al. (2004)            Leutner et al. (2005)
##                                 1                                1
##             Neubert et al. (2014)             Putz-Osterloh (1985)
##                                 1                                1
##               Rigas et al. (2002)       Scherer & Tiemann a (2014)
##                                 1                                1
##        Scherer & Tiemann b (2014)         Sonnleitner et al. (2012)
##                                 1                                1
##          Stadler et al. (In press)    Stadler et al. (Unpublished)
##                                 1                                2
##                 Sub et al. (1991)               Sub et al. (1993)
##                                 1                                1
##                   Wagener (2001)          Wagener & Wittmann (2002)
##                                 4                                1
##              Wirth & Funke (2005)           Wittmann et al. (1996)
##                                 1                                1
##          Wustenberg et al. (2012)         Wustenberg et al. (2014)
##                                 1                                1
```

```r
fit4 <- meta3(y=r, v=v, cluster=Authors, data=Stadler15)
summary(fit4)
```

```
##
## Call:
## meta3(y = r, v = v, cluster = Authors, data = Stadler15)
##
## 95% confidence intervals: z statistic approximation (robust=FALSE)
## Coefficients:
##             Estimate  Std.Error     lbound     ubound z value Pr(>|z|)
## Intercept  0.4348248  0.0323879  0.3713457  0.4983039 13.4255  < 2e-16 ***
## Tau2_2     0.0191179  0.0110196 -0.0024801  0.0407160  1.7349  0.08276 .
## Tau2_3     0.0214491  0.0117158 -0.0015134  0.0444116  1.8308  0.06713 .
## ---
## Signif. codes:  0 '***' 0.001 '**' 0.01 '*' 0.05 '.' 0.1 ' ' 1
##
## Q statistic on the homogeneity of effect sizes: 1155.141
## Degrees of freedom of the Q statistic: 59
## P value of the Q statistic: 0
##
## Heterogeneity indices (based on the estimated Tau2):
##                               Estimate
## I2_2 (Typical v: Q statistic)   0.4528
## I2_3 (Typical v: Q statistic)   0.5080
##
## Number of studies (or clusters): 44
## Number of observed statistics: 60
## Number of estimated parameters: 3
## Degrees of freedom: 57
## -2 log likelihood: -5.44511
## OpenMx status1: 0 ("0" or "1": The optimization is considered fine.
## Other values may indicate problems.)
```

With the IntelligenceMeasure as a moderator

```r
## Display the number of effect sizes per outcome measure
table(Stadler15$IntelligenceMeasure)
```

```
##
## General intelligence            Reasoning
##                   21                   39
```

```r
fit5 <- meta3(y=r, v=v, cluster=Authors, data=Stadler15,
              intercept.constraints=0,
              x=model.matrix(~ -1+IntelligenceMeasure))

## Compare the models with and without the equality constraint
anova(fit5, fit4)
```

```
##                     base            comparison ep  minus2LL df       AIC
## 1 Meta analysis with ML                  <NA>  4 -9.962584 56 -121.9626
## 2 Meta analysis with ML Meta analysis with ML  3 -5.445110 57 -119.4451
##     diffLL diffdf          p
## 1       NA     NA         NA
```

```
## 2 4.517474      1 0.03355032
```

```r
## Slope_1: General intelligence
## Slope_2: Reasoning
summary(fit5)
```

```
##
## Call:
## meta3(y = r, v = v, cluster = Authors, x = model.matrix(~-1 +
##     IntelligenceMeasure), data = Stadler15, intercept.constraints = 0)
##
## 95% confidence intervals: z statistic approximation (robust=FALSE)
## Coefficients:
##            Estimate   Std.Error      lbound      ubound z value  Pr(>|z|)
## Slope_1  0.35088633  0.05009279  0.25270627  0.44906639  7.0047 2.475e-12
## Slope_2  0.48409148  0.03778644  0.41003142  0.55815154 12.8112 < 2.2e-16
## Tau2_2   0.01752965  0.00952992 -0.00114866  0.03620796  1.8394   0.06585
## Tau2_3   0.01868845  0.00996217 -0.00083704  0.03821395  1.8759   0.06066
##
## Slope_1 ***
## Slope_2 ***
## Tau2_2  .
## Tau2_3  .
## ---
## Signif. codes:  0 '***' 0.001 '**' 0.01 '*' 0.05 '.' 0.1 ' ' 1
##
## Q statistic on the homogeneity of effect sizes: 1155.141
## Degrees of freedom of the Q statistic: 59
## P value of the Q statistic: 0
##
## Explained variances (R2):
##                           Level 2 Level 3
## Tau2 (no predictor)      0.019118  0.0214
## Tau2 (with predictors)   0.017530  0.0187
## R2                       0.083077  0.1287
##
## Number of studies (or clusters): 44
## Number of observed statistics: 60
## Number of estimated parameters: 4
## Degrees of freedom: 56
## -2 log likelihood: -9.962584
## OpenMx status1: 0 ("0" or "1": The optimization is considered fine.
## Other values may indicate problems.)
```

Running the analyses in Mplus

Export the data to Mplus

- Multivariate meta-analysis

```r
## Get a copy of the known sampling variances and covariance
my.v <- Nam03[, c("Asthma_v", "AsthmaLRD_cov_05", "LRD_v")]
```

```
## Replace the missing value with 0.
## Since data with missing effect sizes are automatically filtered,
## the imputed value of 0 does not affect the analysis.
my.v[is.na(my.v)] <- 0

## Combine the effect sizes with the known sampling variances and covariance.
my.df <- cbind(Nam03[, c("ID", "Age", "Asthma_logOR", "LRD_logOR")], my.v)

## Display the few cases
head(my.df)

## Write it as a data file
write.table(my.df, "Nam03.dat", na = ".", row.names = FALSE, col.names = FALSE)
```

- Three-level meta-analysis

```
## Get unique numeric labels for the level 3 (study)
## as Mplus only allows numeric data
Study_ID <- with(Stadler15, as.numeric(factor(Authors, level=unique(Authors))))

## Create dummy codes for the Intelligence Measure
GenInt <- ifelse(Stadler15$IntelligenceMeasure=="General intelligence", yes=1, no=0)
Reason <- ifelse(Stadler15$IntelligenceMeasure=="Reasoning", yes=1, no=0)

my.df <- cbind(ID=Stadler15$ID, Study_ID,
               Stadler15[, c("r", "v")], GenInt, Reason)

## Display the few cases
head(my.df, n=8)

## Write it as a data file
write.table(my.df, "Stadler15.dat", na = ".", row.names = FALSE, col.names = FALSE)
```

Multivariate meta-analysis

```
# [Multivariate random-effects meta-analysis](./Nam03a.inp)
# ```
#
# TITLE:    Multivariate random-effects meta-analysis
#
# ! Read the dataset
# DATA: FILE IS Nam03.dat;
#
# ! Variable names in the data
# ! Asthma LRD: effect sizes
# ! Asthma_v Cov_05 LRD_v: known sampling variances and covariance
# VARIABLE: NAMES ID Age Asthma LRD Asthma_v Cov_05 LRD_v;
#
#         ! Missing values are represented by .
#         MISSING ARE .;
#
#         ! Asthma and LRD are used in the analysis.
#         USEVARIABLES ARE Asthma LRD;
```

```
#
#         ! These are the known sampling variances and covariance.
#         CONSTRAINT ARE Asthma_v Cov_05 LRD_v;
#
# MODEL:
#         ! Define two latent variables
#         Lat_Ast BY Asthma;
#         Lat_LRD BY LRD;
#
#         ! Estimate their means
#         [Lat_Ast*];
#         [Lat_LRD*];
#
#         ! Means of the observed variables are fixed at 0.
#         [Asthma@0];
#         [LRD@0];
#
#         ! Label the constraints for the known sampling variances
#         ! and covariances
#         Asthma (L1);
#         LRD (L2);
#         Asthma WITH LRD (L3);
#
#         ! Impose the constraints
#         MODEL CONSTRAINT:
#         L1 = Asthma_v;
#         L2 = LRD_v;
#         L3 = Cov_05;
# ```
#
#
# [Mplus output file](./Nam03a.out)
# ```
#
# Mplus VERSION 8.2 (Linux)
# MUTHEN & MUTHEN
# 02/06/2019   6:31 PM
#
# INPUT INSTRUCTIONS
#
#   TITLE:  Multivariate random-effects meta-analysis
#
#   ! Read the dataset
#   DATA:   FILE IS Nam03.dat;
#
#   ! Variable names in the data
#   ! Asthma LRD: effect sizes
#   ! Asthma_v Cov_05 LRD_v: known sampling variances and covariance
#   VARIABLE: NAMES ID Age Asthma LRD Asthma_v Cov_05 LRD_v;
#
#           ! Missing values are represented by .
#           MISSING ARE .;
#
#           ! Asthma and LRD are used in the analysis.
```

```
#           USEVARIABLES ARE Asthma LRD;
#
#           ! These are the known sampling variances and covariance.
#           CONSTRAINT ARE Asthma_v Cov_05 LRD_v;
#
#   MODEL:
#           ! Define two latent variables
#           Lat_Ast BY Asthma;
#           Lat_LRD BY LRD;
#
#           ! Estimate their means
#           [Lat_Ast*];
#           [Lat_LRD*];
#
#           ! Means of the observed variables are fixed at 0.
#           [Asthma@0];
#           [LRD@0];
#
#           ! Label the constraints for the known sampling variances
#           ! and covariances
#           Asthma (L1);
#           LRD (L2);
#           Asthma WITH LRD (L3);
#
#           ! Impose the constraints
#           MODEL CONSTRAINT:
#           L1 = Asthma_v;
#           L2 = LRD_v;
#           L3 = Cov_05;
#
#
#
# INPUT READING TERMINATED NORMALLY
#
#
#
# Multivariate random-effects meta-analysis
#
# SUMMARY OF ANALYSIS
#
# Number of groups                                                 1
# Number of observations                                          59
#
# Number of dependent variables                                    2
# Number of independent variables                                  0
# Number of continuous latent variables                            2
#
# Observed dependent variables
#
#   Continuous
#    ASTHMA      LRD
#
# Continuous latent variables
#    LAT_AST     LAT_LRD
```

```
#
#
# Estimator                                                    ML
# Information matrix                                     OBSERVED
# Maximum number of iterations                               1000
# Convergence criterion                                 0.500D-04
# Maximum number of steepest descent iterations                20
# Maximum number of iterations for H1                        2000
# Convergence criterion for H1                          0.100D-03
#
# Input data file(s)
#   Nam03.dat
#
# Input data format  FREE
#
#
# SUMMARY OF DATA
#
#      Number of missing data patterns             3
#
#
# COVARIANCE COVERAGE OF DATA
#
# Minimum covariance coverage value   0.100
#
#
#      PROPORTION OF DATA PRESENT
#
#
#            Covariance Coverage
#               ASTHMA        LRD
#               ________      ________
#  ASTHMA         0.542
#  LRD            0.136         0.593
#
#
#
# UNIVARIATE SAMPLE STATISTICS
#
#
#      UNIVARIATE HIGHER-ORDER MOMENT DESCRIPTIVE STATISTICS
#
#         Variable/         Mean/     Skewness/   Minimum/ % with                Percentiles
#        Sample Size      Variance    Kurtosis    Maximum  Min/Max      20%/60%    40%/80%    Median
#
#      ASTHMA                0.268      -0.038      -0.562    3.12%      -0.041      0.191      0.318
#               32.000       0.122       0.110       1.030    3.12%       0.351      0.470
#      LRD                   0.466       1.209      -0.329    2.86%       0.039      0.199      0.285
#               35.000       0.267       0.929       1.933    2.86%       0.419      0.850
#      ASTHMA_V              0.034       3.079       0.000   45.76%       0.000      0.000      0.004
#               59.000       0.004      11.930       0.360    1.69%       0.017      0.053
#      COV_05                0.001       6.762       0.000   86.44%       0.000      0.000      0.000
#               59.000       0.000      46.498       0.045    1.69%       0.000      0.000
#      LRD_V                 0.054       5.551       0.000   40.68%       0.000      0.000      0.005
```

```
#               59.000       0.022      34.387       1.061    1.69%       0.012      0.053
#
#
# THE MODEL ESTIMATION TERMINATED NORMALLY
#
#
#
# MODEL FIT INFORMATION
#
# Number of Free Parameters                        5
#
# Loglikelihood
#
#           H0 Value                         -21.276
#
# Information Criteria
#
#           Akaike (AIC)                      52.552
#           Bayesian (BIC)                    62.940
#           Sample-Size Adjusted BIC          47.216
#             (n* = (n + 2) / 24)
#
#
#
# MODEL RESULTS
#
#                                                     Two-Tailed
#                    Estimate       S.E.  Est./S.E.    P-Value
#
#  LAT_AST  BY
#     ASTHMA            1.000      0.000    999.000    999.000
#
#  LAT_LRD  BY
#     LRD               1.000      0.000    999.000    999.000
#
#  LAT_LRD  WITH
#     LAT_AST           0.056      0.021      2.704      0.007
#
#  ASTHMA   WITH
#     LRD             999.000      0.000    999.000    999.000
#
#  Means
#     LAT_AST           0.268      0.054      4.925      0.000
#     LAT_LRD           0.308      0.054      5.690      0.000
#
#  Intercepts
#     ASTHMA            0.000      0.000    999.000    999.000
#     LRD               0.000      0.000    999.000    999.000
#
#  Variances
#     LAT_AST           0.068      0.030      2.310      0.021
#     LAT_LRD           0.048      0.023      2.094      0.036
#
#  Residual Variances
```

```
#     ASTHMA           999.000      0.000    999.000    999.000
#     LRD              999.000      0.000    999.000    999.000
#
#
# QUALITY OF NUMERICAL RESULTS
#
#      Condition Number for the Information Matrix              0.571E-05
#        (ratio of smallest to largest eigenvalue)
#
#
#      Beginning Time:  18:31:14
#         Ending Time:  18:31:15
#        Elapsed Time:  00:00:01
#
#
#
# MUTHEN & MUTHEN
# 3463 Stoner Ave.
# Los Angeles, CA  90066
#
# Tel: (310) 391-9971
# Fax: (310) 391-8971
# Web: www.StatModel.com
# Support: Support@StatModel.com
#
# Copyright (c) 1998-2018 Muthen & Muthen
# ```
# [Multivariate random-effects meta-analysis with equality constraints](./Nam03b.inp)
# ```
#
# TITLE:    Multivariate random-effects meta-analysis
#         ! Imposing equality constraints and the means and variances
#
# ! Read the dataset
# DATA: FILE IS Nam03.dat;
#
# ! Variable names in the data
# ! Asthma LRD: effect sizes
# ! Asthma_v Cov_05 LRD_v: known sampling variances and covariance
# VARIABLE: NAMES ID Age Asthma LRD Asthma_v Cov_05 LRD_v;
#
#           ! Missing values are represented by .
#         MISSING ARE .;
#
#         ! Asthma and LRD are used in the analysis.
#         USEVARIABLES ARE Asthma LRD;
#
#         ! These are the known sampling variances and covariance.
#         CONSTRAINT ARE Asthma_v Cov_05 LRD_v;
#
# MODEL:
#         ! Define two latent variables
#         Lat_Ast BY Asthma;
```

```
#         Lat_LRD BY LRD;
#
#         ! Latent means are the same
#         [Lat_Ast*] (1);
#         [Lat_LRD*] (1);
#
#         ! Latent variances are the same
#         Lat_Ast* (2);
#         Lat_LRD* (2);
#
#         ! Means of the observed variables are fixed at 0.
#         [Asthma@0];
#         [LRD@0];
#
#         ! Label the constraints for the known sampling variances
#         ! and covariances
#         Asthma (L1);
#         LRD (L2);
#         Asthma WITH LRD (L3);
#
#         ! Impose the constraints
#         MODEL CONSTRAINT:
#         L1 = Asthma_v;
#         L2 = LRD_v;
#         L3 = Cov_05;
# ```
#
#
# [Mplus output file](./Nam03b.out)
# ```
#
# Mplus VERSION 8.2 (Linux)
# MUTHEN & MUTHEN
# 02/06/2019   6:31 PM
#
# INPUT INSTRUCTIONS
#
#   TITLE:  Multivariate random-effects meta-analysis
#           ! Imposing equality constraints and the means and variances
#
#   ! Read the dataset
#   DATA:   FILE IS Nam03.dat;
#
#   ! Variable names in the data
#   ! Asthma LRD: effect sizes
#   ! Asthma_v Cov_05 LRD_v: known sampling variances and covariance
#   VARIABLE: NAMES ID Age Asthma LRD Asthma_v Cov_05 LRD_v;
#
#               ! Missing values are represented by .
#           MISSING ARE .;
#
#           ! Asthma and LRD are used in the analysis.
#           USEVARIABLES ARE Asthma LRD;
#
```

```
#           ! These are the known sampling variances and covariance.
#           CONSTRAINT ARE Asthma_v Cov_05 LRD_v;
#
#   MODEL:
#           ! Define two latent variables
#           Lat_Ast BY Asthma;
#           Lat_LRD BY LRD;
#
#           ! Latent means are the same
#           [Lat_Ast*] (1);
#           [Lat_LRD*] (1);
#
#           ! Latent variances are the same
#           Lat_Ast* (2);
#           Lat_LRD* (2);
#
#           ! Means of the observed variables are fixed at 0.
#           [Asthma@0];
#           [LRD@0];
#
#           ! Label the constraints for the known sampling variances
#           ! and covariances
#           Asthma (L1);
#           LRD (L2);
#           Asthma WITH LRD (L3);
#
#           ! Impose the constraints
#           MODEL CONSTRAINT:
#           L1 = Asthma_v;
#           L2 = LRD_v;
#           L3 = Cov_05;
#
#
#
# INPUT READING TERMINATED NORMALLY
#
#
#
# Multivariate random-effects meta-analysis
#
# SUMMARY OF ANALYSIS
#
# Number of groups                                                 1
# Number of observations                                          59
#
# Number of dependent variables                                    2
# Number of independent variables                                  0
# Number of continuous latent variables                            2
#
# Observed dependent variables
#
#   Continuous
#    ASTHMA      LRD
#
```

```
# Continuous latent variables
#    LAT_AST     LAT_LRD
#
#
# Estimator                                                      ML
# Information matrix                                       OBSERVED
# Maximum number of iterations                                 1000
# Convergence criterion                                   0.500D-04
# Maximum number of steepest descent iterations                  20
# Maximum number of iterations for H1                          2000
# Convergence criterion for H1                            0.100D-03
#
# Input data file(s)
#   Nam03.dat
#
# Input data format  FREE
#
#
# SUMMARY OF DATA
#
#      Number of missing data patterns             3
#
#
# COVARIANCE COVERAGE OF DATA
#
# Minimum covariance coverage value   0.100
#
#
#      PROPORTION OF DATA PRESENT
#
#
#            Covariance Coverage
#               ASTHMA        LRD
#               --------      --------
#  ASTHMA         0.542
#  LRD            0.136         0.593
#
#
#
# UNIVARIATE SAMPLE STATISTICS
#
#
#      UNIVARIATE HIGHER-ORDER MOMENT DESCRIPTIVE STATISTICS
#
#         Variable/         Mean/     Skewness/   Minimum/ % with                Percentiles
#        Sample Size      Variance    Kurtosis    Maximum  Min/Max      20%/60%    40%/80%    Median
#
#      ASTHMA               0.268      -0.038      -0.562    3.12%      -0.041      0.191      0.318
#              32.000       0.122       0.110       1.030    3.12%       0.351      0.470
#      LRD                  0.466       1.209      -0.329    2.86%       0.039      0.199      0.285
#              35.000       0.267       0.929       1.933    2.86%       0.419      0.850
#      ASTHMA_V             0.034       3.079       0.000   45.76%       0.000      0.000      0.004
#              59.000       0.004      11.930       0.360    1.69%       0.017      0.053
#      COV_05               0.001       6.762       0.000   86.44%       0.000      0.000      0.000
```

```
#              59.000       0.000      46.498       0.045    1.69%       0.000      0.000
#      LRD_V                0.054       5.551       0.000   40.68%       0.000      0.000      0.005
#              59.000       0.022      34.387       1.061    1.69%       0.012      0.053
#
#
# THE MODEL ESTIMATION TERMINATED NORMALLY
#
#
#
# MODEL FIT INFORMATION
#
# Number of Free Parameters                        3
#
# Loglikelihood
#
#          H0 Value                          -22.664
#
# Information Criteria
#
#          Akaike (AIC)                       51.327
#          Bayesian (BIC)                     57.560
#          Sample-Size Adjusted BIC           48.126
#            (n* = (n + 2) / 24)
#
#
#
# MODEL RESULTS
#
#                                                    Two-Tailed
#                    Estimate       S.E.  Est./S.E.    P-Value
#
#  LAT_AST  BY
#     ASTHMA             1.000      0.000    999.000    999.000
#
#  LAT_LRD  BY
#     LRD                1.000      0.000    999.000    999.000
#
#  LAT_LRD  WITH
#     LAT_AST            0.050      0.021      2.373      0.018
#
#  ASTHMA   WITH
#     LRD              999.000      0.000    999.000    999.000
#
#  Means
#     LAT_AST            0.293      0.043      6.817      0.000
#     LAT_LRD            0.293      0.043      6.817      0.000
#
#  Intercepts
#     ASTHMA             0.000      0.000    999.000    999.000
#     LRD                0.000      0.000    999.000    999.000
#
#  Variances
#     LAT_AST            0.055      0.020      2.761      0.006
#     LAT_LRD            0.055      0.020      2.761      0.006
```

```
#
#  Residual Variances
#     ASTHMA           999.000      0.000    999.000    999.000
#     LRD              999.000      0.000    999.000    999.000
#
#
# QUALITY OF NUMERICAL RESULTS
#
#      Condition Number for the Information Matrix              0.148E-04
#        (ratio of smallest to largest eigenvalue)
#
#
#      Beginning Time:  18:31:17
#         Ending Time:  18:31:17
#        Elapsed Time:  00:00:00
#
#
#
# MUTHEN & MUTHEN
# 3463 Stoner Ave.
# Los Angeles, CA  90066
#
# Tel: (310) 391-9971
# Fax: (310) 391-8971
# Web: www.StatModel.com
# Support: Support@StatModel.com
#
# Copyright (c) 1998-2018 Muthen & Muthen
# ```
# [Multivariate mixed-effects meta-analysis](./Nam03c.inp)
# ```
#
# TITLE:    Multivariate mixed-effects meta-analysis with age as a moderator
#
# ! Read the dataset
# DATA: FILE IS Nam03.dat;
#
# ! Variable names in the data
# ! Asthma LRD: effect sizes
# ! Asthma_v Cov_05 LRD_v: known sampling variances and covariance
# VARIABLE: NAMES ID Age Asthma LRD Asthma_v Cov_05 LRD_v;
#
#         ! Missing values are represented by .
#         MISSING ARE .;
#
#         ! Asthma and LRD are used in the analysis.
#         ! Age is a moderator.
#         USEVARIABLES ARE Asthma LRD Age;
#
#         ! These are the known sampling variances and covariance.
#         CONSTRAINT ARE Asthma_v Cov_05 LRD_v;
#
#         DEFINE:
```

```
#         ! Center Age before the analysis
#         CENTER Age (GRANDMEAN);
#
# MODEL:
#         ! Define two latent variables
#         Lat_Ast BY Asthma;
#         Lat_LRD BY LRD;
#
#         ! Fix the covariance of between the random effects at 0
#         ! as there is not enough data.
#         Lat_Ast WITH Lat_LRD@0;
#
#         ! Estimate their means
#         [Lat_Ast*];
#         [Lat_LRD*];
#
#         ! Means of the observed variables are fixed at 0.
#         [Asthma@0];
#         [LRD@0];
#
#         ! Label the constraints for the known sampling variances
#         ! and covariances
#         Asthma (L1);
#         LRD (L2);
#         Asthma WITH LRD (L3);
#
#         ! Regress the latent effect sizes on Age
#         Lat_Ast Lat_LRD ON Age;
#
#         ! Estimate the mean of Age
#         [Age*];
#
#         ! Estimate the variance of Age
#         Age*;
#
#         ! Impose the constraints
#         MODEL CONSTRAINT:
#         L1 = Asthma_v;
#         L2 = LRD_v;
#         L3 = Cov_05;
# ```
#
#
# [Mplus output file](./Nam03c.out)
# ```
#
# Mplus VERSION 8.2 (Linux)
# MUTHEN & MUTHEN
# 02/06/2019   6:31 PM
#
# INPUT INSTRUCTIONS
#
#   TITLE:  Multivariate mixed-effects meta-analysis with age as a moderator
#
```

```
#   ! Read the dataset
#   DATA:   FILE IS Nam03.dat;
#
#   ! Variable names in the data
#   ! Asthma LRD: effect sizes
#   ! Asthma_v Cov_05 LRD_v: known sampling variances and covariance
#   VARIABLE: NAMES ID Age Asthma LRD Asthma_v Cov_05 LRD_v;
#
#           ! Missing values are represented by .
#           MISSING ARE .;
#
#           ! Asthma and LRD are used in the analysis.
#           ! Age is a moderator.
#           USEVARIABLES ARE Asthma LRD Age;
#
#           ! These are the known sampling variances and covariance.
#           CONSTRAINT ARE Asthma_v Cov_05 LRD_v;
#
#           DEFINE:
#           ! Center Age before the analysis
#           CENTER Age (GRANDMEAN);
#
#   MODEL:
#           ! Define two latent variables
#           Lat_Ast BY Asthma;
#           Lat_LRD BY LRD;
#
#           ! Fix the covariance of between the random effects at 0
#           ! as there is not enough data.
#           Lat_Ast WITH Lat_LRD@0;
#
#           ! Estimate their means
#           [Lat_Ast*];
#           [Lat_LRD*];
#
#           ! Means of the observed variables are fixed at 0.
#           [Asthma@0];
#           [LRD@0];
#
#           ! Label the constraints for the known sampling variances
#           ! and covariances
#           Asthma (L1);
#           LRD (L2);
#           Asthma WITH LRD (L3);
#
#           ! Regress the latent effect sizes on Age
#           Lat_Ast Lat_LRD ON Age;
#
#           ! Estimate the mean of Age
#           [Age*];
#
#           ! Estimate the variance of Age
#           Age*;
#
```

```
#           ! Impose the constraints
#           MODEL CONSTRAINT:
#           L1 = Asthma_v;
#           L2 = LRD_v;
#           L3 = Cov_05;
#
#
#
# INPUT READING TERMINATED NORMALLY
#
#
#
# Multivariate mixed-effects meta-analysis with age as a moderator
#
# SUMMARY OF ANALYSIS
#
# Number of groups                                                 1
# Number of observations                                          59
#
# Number of dependent variables                                    2
# Number of independent variables                                  1
# Number of continuous latent variables                            2
#
# Observed dependent variables
#
#   Continuous
#    ASTHMA      LRD
#
# Observed independent variables
#    AGE
#
# Continuous latent variables
#    LAT_AST     LAT_LRD
#
# Variables with special functions
#
#   Centering (GRANDMEAN)
#    AGE
#
#
# Estimator                                                       ML
# Information matrix                                        OBSERVED
# Maximum number of iterations                                  1000
# Convergence criterion                                    0.500D-04
# Maximum number of steepest descent iterations                   20
# Maximum number of iterations for H1                           2000
# Convergence criterion for H1                             0.100D-03
#
# Input data file(s)
#   Nam03.dat
#
# Input data format  FREE
#
#
```

```
# SUMMARY OF DATA
#
#      Number of missing data patterns             3
#
#
# COVARIANCE COVERAGE OF DATA
#
# Minimum covariance coverage value   0.100
#
#
#      PROPORTION OF DATA PRESENT
#
#
#            Covariance Coverage
#               ASTHMA        LRD           AGE
#               --------      --------      --------
#  ASTHMA         0.542
#  LRD            0.136         0.593
#  AGE            0.542         0.593         1.000
#
#
#
# UNIVARIATE SAMPLE STATISTICS
#
#
#      UNIVARIATE HIGHER-ORDER MOMENT DESCRIPTIVE STATISTICS
#
#          Variable/         Mean/     Skewness/   Minimum/ % with                Percentiles
#         Sample Size      Variance    Kurtosis    Maximum  Min/Max      20%/60%    40%/80%    Median
#
#      ASTHMA                 0.268      -0.038      -0.562    3.12%      -0.041      0.191      0.318
#               32.000        0.122       0.110       1.030    3.12%       0.351      0.470
#      LRD                    0.466       1.209      -0.329    2.86%       0.039      0.199      0.285
#               35.000        0.267       0.929       1.933    2.86%       0.419      0.850
#      AGE                    0.000      -0.106      -6.403    3.39%      -4.903      0.097      1.097
#               59.000       15.580      -0.720       9.397    1.69%       1.697      2.597
#      ASTHMA_V               0.034       3.079       0.000   45.76%       0.000      0.000      0.004
#               59.000        0.004      11.930       0.360    1.69%       0.017      0.053
#      COV_05                 0.001       6.762       0.000   86.44%       0.000      0.000      0.000
#               59.000        0.000      46.498       0.045    1.69%       0.000      0.000
#      LRD_V                  0.054       5.551       0.000   40.68%       0.000      0.000      0.005
#               59.000        0.022      34.387       1.061    1.69%       0.012      0.053
#
#
# THE MODEL ESTIMATION TERMINATED NORMALLY
#
#
#
# MODEL FIT INFORMATION
#
# Number of Free Parameters                        8
#
# Loglikelihood
#
```

```
#           H0 Value                        -184.142
#
# Information Criteria
#
#           Akaike (AIC)                     384.284
#           Bayesian (BIC)                   400.904
#           Sample-Size Adjusted BIC         375.747
#             (n* = (n + 2) / 24)
#
#
#
# MODEL RESULTS
#
#                                                    Two-Tailed
#                    Estimate       S.E.  Est./S.E.    P-Value
#
#  LAT_AST  BY
#     ASTHMA            1.000      0.000    999.000    999.000
#
#  LAT_LRD  BY
#     LRD               1.000      0.000    999.000    999.000
#
#  LAT_AST  ON
#     AGE              -0.038      0.016     -2.444      0.015
#
#  LAT_LRD  ON
#     AGE              -0.024      0.009     -2.531      0.011
#
#  LAT_AST  WITH
#     LAT_LRD           0.000      0.000    999.000    999.000
#
#  ASTHMA   WITH
#     LRD             999.000      0.000    999.000    999.000
#
#  Means
#     AGE               0.000      0.514      0.000      1.000
#
#  Intercepts
#     ASTHMA            0.000      0.000    999.000    999.000
#     LRD               0.000      0.000    999.000    999.000
#     LAT_AST           0.268      0.049      5.509      0.000
#     LAT_LRD           0.273      0.047      5.753      0.000
#
#  Variances
#     AGE              15.580      2.868      5.431      0.000
#
#  Residual Variances
#     ASTHMA          999.000      0.000    999.000    999.000
#     LRD             999.000      0.000    999.000    999.000
#     LAT_AST           0.028      0.014      1.967      0.049
#     LAT_LRD           0.030      0.016      1.903      0.057
#
#
# QUALITY OF NUMERICAL RESULTS
```

```
#
#      Condition Number for the Information Matrix              0.850E-04
#        (ratio of smallest to largest eigenvalue)
#
#
#      Beginning Time:  18:31:19
#         Ending Time:  18:31:20
#        Elapsed Time:  00:00:01
#
#
#
# MUTHEN & MUTHEN
# 3463 Stoner Ave.
# Los Angeles, CA  90066
#
# Tel: (310) 391-9971
# Fax: (310) 391-8971
# Web: www.StatModel.com
# Support: Support@StatModel.com
#
# Copyright (c) 1998-2018 Muthen & Muthen
# ```
```

Three-level meta-analysis

```
# [Three-level random-effects meta-analysis](./Stadler15a.inp)
# ```
#
# TITLE:    Three-level random-effects meta-analysis
#
# ! Read the dataset
# DATA: FILE IS Stadler15.dat;
#
# ! Variable names in the dataset
# ! ID: Level 2 label
# ! Study: Level 3 label
# ! r: effect size (correlation)
# ! v: known sampling variance of r
# ! GenInt: Dummy code for Intelligence Measure (General Intelligence)
# ! Reason: Dummy code for Intelligence Measure (Reasoning)
# VARIABLE: NAMES ID Study r v GenInt Reason;
#
#         ! w2 is the square root of v
#         USEVARIABLES ARE r w2;
#         ! Define level 2 and level 3
#         CLUSTER = Study ID;
#         ! Define within level (level 2) variables without between (level 3)
#         WITHIN = r w2;
#
#         ! Transform the effect sizes
#         DEFINE: w2 = SQRT(v**(-1));
#         r = w2*r;
#
```

```
# ! Use three-level modeling
# ! Activate random slope function
# ANALYSIS: TYPE=THREELEVEL RANDOM;
#         ESTIMATOR = ML;
#
# MODEL:    %WITHIN%
#         ! Mean and variance of the transformed variable are fixed after the transformation
#         [r@0.0];
#         r@1.0;
#
#         ! Define random slope
#         f | r ON w2;
#
#         ! Level 2 variance
#         %BETWEEN ID%
#         f*;
#
#         ! Level 3 variance
#         %BETWEEN Study%
#         f*;
# ```
#
#
# [Mplus output file](./Stadler15a.out)
# ```
#
# Mplus VERSION 8.2 (Linux)
# MUTHEN & MUTHEN
# 05/27/2019  10:58 AM
#
# INPUT INSTRUCTIONS
#
#   TITLE:  Three-level random-effects meta-analysis
#
#   ! Read the dataset
#   DATA:   FILE IS Stadler15.dat;
#
#   ! Variable names in the dataset
#   ! ID: Level 2 label
#   ! Study: Level 3 label
#   ! r: effect size (correlation)
#   ! v: known sampling variance of r
#   ! GenInt: Dummy code for Intelligence Measure (General Intelligence)
#   ! Reason: Dummy code for Intelligence Measure (Reasoning)
#   VARIABLE: NAMES ID Study r v GenInt Reason;
#
#           ! w2 is the square root of v
#           USEVARIABLES ARE r w2;
#           ! Define level 2 and level 3
#           CLUSTER = Study ID;
#           ! Define within level (level 2) variables without between (level 3)
#           WITHIN = r w2;
#
#           ! Transform the effect sizes
```

```
#           DEFINE: w2 = SQRT(v**(-1));
#           r = w2*r;
#
#   ! Use three-level modeling
#   ! Activate random slope function
#   ANALYSIS: TYPE=THREELEVEL RANDOM;
#           ESTIMATOR = ML;
#
#   MODEL:  %WITHIN%
#           ! Mean and variance of the transformed variable are fixed after the transformation
#           [r@0.0];
#           r@1.0;
#
#           ! Define random slope
#           f | r ON w2;
#
#           ! Level 2 variance
#           %BETWEEN ID%
#           f*;
#
#           ! Level 3 variance
#           %BETWEEN Study%
#           f*;
#
#
#
# *** WARNING in MODEL command
#   Variable on the left-hand side of an ON statement in a | statement is a
#   WITHIN variable.  The intercept for this variable is not random.
#   Variable:  R
#    1 WARNING(S) FOUND IN THE INPUT INSTRUCTIONS
#
#
#
# Three-level random-effects meta-analysis
#
# SUMMARY OF ANALYSIS
#
# Number of groups                                                 1
# Number of observations                                          60
#
# Number of dependent variables                                    1
# Number of independent variables                                  1
# Number of continuous latent variables                            1
#
# Observed dependent variables
#
#   Continuous
#    R
#
# Observed independent variables
#    W2
#
# Continuous latent variables
```

```
#    F
#
# Variables with special functions
#
#   Cluster variables     STUDY     ID
#
#   Within variables
#    R           W2
#
#
# Estimator                                                       ML
# Information matrix                                        OBSERVED
# Maximum number of iterations                                   100
# Convergence criterion                                    0.100D-05
# Maximum number of EM iterations                                500
# Convergence criteria for the EM algorithm
#   Loglikelihood change                                   0.100D-02
#   Relative loglikelihood change                          0.100D-05
#   Derivative                                             0.100D-03
# Minimum variance                                         0.100D-03
# Maximum number of steepest descent iterations                   20
# Maximum number of iterations for H1                           2000
# Convergence criterion for H1                             0.100D-02
# Optimization algorithm                                         EMA
#
# Input data file(s)
#   Stadler15.dat
# Input data format  FREE
#
#
# SUMMARY OF DATA
#
#      Number of ID clusters                    60
#      Number of STUDY clusters                 44
#
#
#
# UNIVARIATE SAMPLE STATISTICS
#
#
#      UNIVARIATE HIGHER-ORDER MOMENT DESCRIPTIVE STATISTICS
#
#         Variable/         Mean/     Skewness/   Minimum/ % with                Percentiles
#        Sample Size      Variance    Kurtosis    Maximum  Min/Max      20%/60%    40%/80%    Median
#
#      R                     9.555       2.689      -1.234    1.67%       1.425      2.760      3.769
#             60.000       212.394       7.580      72.912    1.67%       5.428     13.103
#      W2                   17.860       2.504       3.991    1.67%       6.187      8.616      9.714
#             60.000       353.168       6.858     100.071    1.67%      14.408     23.429
#
#
# THE MODEL ESTIMATION TERMINATED NORMALLY
#
#
```

```
#
# MODEL FIT INFORMATION
#
# Number of Free Parameters                        3
#
# Loglikelihood
#
#          H0 Value                         -148.458
#
# Information Criteria
#
#          Akaike (AIC)                      302.915
#          Bayesian (BIC)                    309.198
#          Sample-Size Adjusted BIC          299.763
#            (n* = (n + 2) / 24)
#
#
#
# MODEL RESULTS
#
#                                                    Two-Tailed
#                    Estimate       S.E.  Est./S.E.    P-Value
#
# Within Level
#
#  Intercepts
#     R                 0.000      0.000    999.000    999.000
#
#  Residual Variances
#     R                 1.000      0.000    999.000    999.000
#
# Between ID Level
#
#  Variances
#     F                 0.019      0.011      1.735      0.083
#
# Between STUDY Level
#
#  Means
#     F                 0.435      0.032     13.426      0.000
#
#  Variances
#     F                 0.021      0.012      1.831      0.067
#
#
# QUALITY OF NUMERICAL RESULTS
#
#      Condition Number for the Information Matrix              0.425E-01
#        (ratio of smallest to largest eigenvalue)
#
#
#      Beginning Time:  10:58:17
#         Ending Time:  10:58:17
#        Elapsed Time:  00:00:00
```

```
#
#
#
# MUTHEN & MUTHEN
# 3463 Stoner Ave.
# Los Angeles, CA  90066
#
# Tel: (310) 391-9971
# Fax: (310) 391-8971
# Web: www.StatModel.com
# Support: Support@StatModel.com
#
# Copyright (c) 1998-2018 Muthen & Muthen
# ```
# [Three-level mixed-effects meta-analysis](./Stadler15b.inp)
# ```
#
# TITLE:    Three-level mixed-effects meta-analysis
#
# ! Read the dataset
# DATA: FILE IS Stadler15.dat;
#
# ! Variable names in the dataset
# ! ID: Level 2 label
# ! Study: Level 3 label
# ! r: effect size (correlation)
# ! v: known sampling variance of r
# ! GenInt: Dummy code for Intelligence Measure (General Intelligence)
# ! Reason: Dummy code for Intelligence Measure (Reasoning)
# VARIABLE: NAMES ID Study r v GenInt Reason;
#
#         ! w2 is the square root of v
#         USEVARIABLES ARE r GenInt Reason w2;
#         ! Define level 2 and level 3
#         CLUSTER = Study ID;
#         WITHIN = r w2;
#         ! Define level 3 variables
#         BETWEEN = (ID) GenInt Reason;
#
#         ! Transform the effect sizes.
#         DEFINE: w2 = SQRT(v**(-1));
#         r = w2*r;
#
# ! Use three-level modeling
# ! Activate random slope function
# ANALYSIS: TYPE=THREELEVEL RANDOM;
#         ESTIMATOR = ML;
#
# MODEL:    %WITHIN%
#         ! Mean and variance of the transformed variable are fixed
#         [r@0.0];
#         r@1.0;
#
```

```
#         ! Define random slope
#         f | r ON w2;
#
#         ! Level 2 variance
#         %BETWEEN ID%
#         f*;
#
#         ! No intercept for the categorical modeator
#         [f@0.0];
#         ! Add the moderators
#       f ON GenInt Reason;
#
#         ! Level 3 variance
#         %BETWEEN Study%
#         f*;
#         ! No intercept for the categorical modeator
#         [f@0.0];
# ```
#
#
# [Mplus output file](./Stadler15b.out)
# ```
#
# Mplus VERSION 8.2 (Linux)
# MUTHEN & MUTHEN
# 05/27/2019  11:39 AM
#
# INPUT INSTRUCTIONS
#
#   TITLE:  Three-level mixed-effects meta-analysis
#
#   ! Read the dataset
#   DATA:   FILE IS Stadler15.dat;
#
#   ! Variable names in the dataset
#   ! ID: Level 2 label
#   ! Study: Level 3 label
#   ! r: effect size (correlation)
#   ! v: known sampling variance of r
#   ! GenInt: Dummy code for Intelligence Measure (General Intelligence)
#   ! Reason: Dummy code for Intelligence Measure (Reasoning)
#   VARIABLE: NAMES ID Study r v GenInt Reason;
#
#           ! w2 is the square root of v
#           USEVARIABLES ARE r GenInt Reason w2;
#           ! Define level 2 and level 3
#           CLUSTER = Study ID;
#           WITHIN = r w2;
#           ! Define level 3 variables
#           BETWEEN = (ID) GenInt Reason;
#
#           ! Transform the effect sizes.
#           DEFINE: w2 = SQRT(v**(-1));
#           r = w2*r;
```

```
#
#   ! Use three-level modeling
#   ! Activate random slope function
#   ANALYSIS: TYPE=THREELEVEL RANDOM;
#           ESTIMATOR = ML;
#
#   MODEL:  %WITHIN%
#           ! Mean and variance of the transformed variable are fixed
#           [r@0.0];
#           r@1.0;
#
#           ! Define random slope
#           f | r ON w2;
#
#           ! Level 2 variance
#           %BETWEEN ID%
#           f*;
#
#           ! No intercept for the categorical modeator
#           [f@0.0];
#           ! Add the moderators
#           f ON GenInt Reason;
#
#           ! Level 3 variance
#           %BETWEEN Study%
#           f*;
#           ! No intercept for the categorical modeator
#           [f@0.0];
#
#
#
# *** WARNING in MODEL command
#   Variable on the left-hand side of an ON statement in a | statement is a
#   WITHIN variable.  The intercept for this variable is not random.
#   Variable:  R
#    1 WARNING(S) FOUND IN THE INPUT INSTRUCTIONS
#
#
#
# Three-level mixed-effects meta-analysis
#
# SUMMARY OF ANALYSIS
#
# Number of groups                                                 1
# Number of observations                                          60
#
# Number of dependent variables                                    1
# Number of independent variables                                  3
# Number of continuous latent variables                            1
#
# Observed dependent variables
#
#   Continuous
#    R
```

```
#
# Observed independent variables
#    GENINT      REASON      W2
#
# Continuous latent variables
#    F
#
# Variables with special functions
#
#   Cluster variables     STUDY     ID
#
#   Within variables
#    R           W2
#
#   Level 2 between variables
#    GENINT      REASON
#
#
# Estimator                                                    ML
# Information matrix                                     OBSERVED
# Maximum number of iterations                                100
# Convergence criterion                                 0.100D-05
# Maximum number of EM iterations                             500
# Convergence criteria for the EM algorithm
#   Loglikelihood change                                0.100D-02
#   Relative loglikelihood change                       0.100D-05
#   Derivative                                          0.100D-03
# Minimum variance                                      0.100D-03
# Maximum number of steepest descent iterations                20
# Maximum number of iterations for H1                        2000
# Convergence criterion for H1                          0.100D-02
# Optimization algorithm                                      EMA
#
# Input data file(s)
#   Stadler15.dat
# Input data format  FREE
#
#
# SUMMARY OF DATA
#
#      Number of ID clusters                    60
#      Number of STUDY clusters                 44
#
#
#
# UNIVARIATE SAMPLE STATISTICS
#
#
#      UNIVARIATE HIGHER-ORDER MOMENT DESCRIPTIVE STATISTICS
#
#          Variable/         Mean/     Skewness/   Minimum/ % with                Percentiles
#         Sample Size      Variance    Kurtosis    Maximum  Min/Max      20%/60%    40%/80%    Median
#
#      R                     9.555       2.689      -1.234    1.67%       1.425      2.760      3.769
```

```
#              60.000     212.394       7.580      72.912    1.67%       5.428     13.103
#      GENINT                0.350       0.629       0.000   65.00%       0.000      0.000      0.000
#              60.000       0.227      -1.604       1.000   35.00%       0.000      1.000
#      REASON                0.650      -0.629       0.000   35.00%       0.000      1.000      1.000
#              60.000       0.227      -1.604       1.000   65.00%       1.000      1.000
#      W2                   17.860       2.504       3.991    1.67%       6.187      8.616      9.714
#              60.000     353.168       6.858     100.071    1.67%      14.408     23.429
#
#
# THE MODEL ESTIMATION TERMINATED NORMALLY
#
#
#
# MODEL FIT INFORMATION
#
# Number of Free Parameters                        4
#
# Loglikelihood
#
#           H0 Value                        -146.199
#
# Information Criteria
#
#           Akaike (AIC)                     300.398
#           Bayesian (BIC)                   308.775
#           Sample-Size Adjusted BIC         296.194
#             (n* = (n + 2) / 24)
#
#
#
# MODEL RESULTS
#
#                                                    Two-Tailed
#                    Estimate       S.E.  Est./S.E.    P-Value
#
# Within Level
#
#  Intercepts
#     R                 0.000      0.000    999.000    999.000
#
#  Residual Variances
#     R                 1.000      0.000    999.000    999.000
#
# Between ID Level
#
#  F          ON
#     GENINT            0.351      0.050      7.005      0.000
#     REASON            0.484      0.038     12.811      0.000
#
#  Intercepts
#     F                 0.000      0.000    999.000    999.000
#
#  Residual Variances
#     F                 0.018      0.010      1.839      0.066
```

```
#
# Between STUDY Level
#
#  Means
#     F                  0.000      0.000    999.000    999.000
#
#  Variances
#     F                  0.019      0.010      1.876      0.061
#
#
# QUALITY OF NUMERICAL RESULTS
#
#      Condition Number for the Information Matrix              0.129E-01
#        (ratio of smallest to largest eigenvalue)
#
#
#      Beginning Time:  11:39:29
#         Ending Time:  11:39:29
#        Elapsed Time:  00:00:00
#
#
#
# MUTHEN & MUTHEN
# 3463 Stoner Ave.
# Los Angeles, CA  90066
#
# Tel: (310) 391-9971
# Fax: (310) 391-8971
# Web: www.StatModel.com
# Support: Support@StatModel.com
#
# Copyright (c) 1998-2018 Muthen & Muthen
# ```
```

```r
cat("\n\n")
```

```r
## Settings of the computing environment
sessionInfo()
```

```
## R version 3.6.1 (2019-07-05)
## Platform: x86_64-pc-linux-gnu (64-bit)
## Running under: Linux Mint 19.1
##
## Matrix products: default
## BLAS:   /usr/lib/x86_64-linux-gnu/openblas/libblas.so.3
## LAPACK: /usr/lib/x86_64-linux-gnu/libopenblasp-r0.2.20.so
##
## locale:
##  [1] LC_CTYPE=en_SG.UTF-8       LC_NUMERIC=C
##  [3] LC_TIME=en_SG.UTF-8        LC_COLLATE=en_SG.UTF-8
##  [5] LC_MONETARY=en_SG.UTF-8    LC_MESSAGES=en_SG.UTF-8
##  [7] LC_PAPER=en_SG.UTF-8       LC_NAME=C
##  [9] LC_ADDRESS=C               LC_TELEPHONE=C
## [11] LC_MEASUREMENT=en_SG.UTF-8 LC_IDENTIFICATION=C
##
```

```
## attached base packages:
## [1] stats     graphics  grDevices utils     datasets  methods   base
##
## other attached packages:
## [1] metafor_2.1-0   Matrix_1.2-17   metaSEM_1.2.2.1 OpenMx_2.13.2
##
## loaded via a namespace (and not attached):
##  [1] Rcpp_1.0.1      knitr_1.23      magrittr_1.5    MASS_7.3-51.4
##  [5] mnormt_1.5-5    pbivnorm_0.6.0  ellipse_0.4.1   lattice_0.20-38
##  [9] stringr_1.4.0   tools_3.6.1     parallel_3.6.1  grid_3.6.1
## [13] nlme_3.1-140    xfun_0.8        htmltools_0.3.6 yaml_2.2.0
## [17] digest_0.6.20   lavaan_0.6-4    evaluate_0.14   rmarkdown_1.13
## [21] stringi_1.4.3   compiler_3.6.1  stats4_3.6.1    mvtnorm_1.0-11
```
